# The dynamic lives of osseous points from Late Palaeolithic/Early Mesolithic Doggerland: A detailed functional study of barbed and unbarbed points from the Dutch North Sea

**DOI:** 10.1371/journal.pone.0288629

**Published:** 2023-08-02

**Authors:** Alessandro Aleo, Paul R. B. Kozowyk, Liliana I. Baron, Annelou van Gijn, Geeske H. J. Langejans

**Affiliations:** 1 Faculty of Mechanical, Maritime and Materials Engineering, Department of Materials Science and Engineering, Delft University of Technology, Delft, the Netherlands; 2 Faculty of Archaeology, Department of Archaeological Sciences, Leiden University, Leiden, the Netherlands; 3 Faculty of Applied Science, Department of Chemical Engineering, Delft University of Technology, Delft, the Netherlands; 4 Palaeo-Research Institute, University of Johannesburg, Johannesburg, South Africa; University of Glasgow College of Arts, UNITED KINGDOM

## Abstract

Osseous barbed and unbarbed points are commonly recovered from the Dutch North Sea and other Mesolithic sites of northern Europe. Interpreted as elements of projectile weaponry, barbed points are considered by archaeologists to be a technological innovation in the hunting equipment of hunter-gatherers. However, debate about their exact use and identification of the targeted prey species is still ongoing. To shed light on the function of these tools, we analysed a sample of 17 artefacts from the Netherlands with a multi-disciplinary approach encompassing morphometric, functional, and chemical analysis. ^14^C-AMS dating yielded the oldest date for a barbed point from the Dutch coast (⁓13000 cal. BP). The observation of microwear traces preserved on the tools provides solid evidence to interpret the function of barbed and unbarbed points. We show that there were two distinct tool categories. 1) Barbed points hafted with birch tar and animal or vegetal binding were likely projectile tips for terrestrial and aquatic hunting. We provide strong clues to support the link between small barbed points and fishing using wear traces. 2) Points without barbs served as perforators for animal hides. Our results highlight the importance of use-wear and residue analysis to reconstruct prehistoric hunting activities. The functional interpretation of projectile points must also rely on microwear traces and not merely on the association with faunal remains, historical sources, and ethnographic comparisons.

## 1. Introduction

The appearance of barbed points in the archaeological record can be seen as a major innovation in the hunting equipment of hunter-gatherers linked to new predatory strategies [1]. In Europe, the oldest self-barbed point dates to the end of the Gravettian [2]. The use of barbed osseous points spread during the Magdalenian [3] and continued, in different forms, in the following technocomplexes. Barbed points are also widespread and typical artefacts from the Mesolithic. They were found in large amounts in Mesolithic contexts of northeaster Germany [4,5], southern Scandinavia [6], western Russia [7], southern Baltic [8,9], the Netherlands [10], and Great Britain [11]. Differences in technology and morphology that characterise barbed weaponry are linked to cultural traditions. Although techno-functional differences exist, their frequency in Mesolithic sites of northern Europe proves that these artefacts were part of a common technological practice shared by Mesolithic people.

For nearly a century there has been debate concerning the function of osseous barbed points. Their function has been inferred based on direct and indirect association with faunal remains, ethnographic comparisons, and sporadically with use-wear studies that are mostly limited to fracture patterns and macrowear traces. Clark [12,13] first interpreted osseous points as fishing gear, while others [e.g., 5, 14 and references therein] have argued that these tools were used on both terrestrial and aquatic prey. No direct and exclusive association with fish has been found in the archaeological record so far [see 5]. However, the indirect association of barbed points and fish bones, mostly from pike, is documented at numerous sites including Abri of Liesbergmühle VI, Switzerland [15], Odmut, Montenegro [16], Kunda, Estonia [14] and the Trans-Urals [17]. At the Late Mesolithic site of Abri of Liesbergmühle, for instance, many osseous points were found in association with fish remains, which constitute around 20% of the faunal assemblage [15]. Large barbed points were recovered in direct association with elk remains at High Furlong, England [18] and Tåderup, Denmark [13]. These finds document the use of barbed tips for hunting large herbivores.

Historical and ethnographic accounts have shown a varied rather than specialised use of barbed points. Based on ethnographic accounts, detachable harpoons are specialised hunting tools intended for marine mammals and hunting in aquatic environments [19,20]. Undetachable ones (or barbed points) are for fishing, for hunting birds, otters, land mammals, and even for war [1,19,20]. Ethnography can provide possible explanations or be useful in forming hypotheses about ancient use, but cannot be relied upon on its own. Thus far morphometric studies, fauna associations, and analogies have not resolved the function of Mesolithic osseous barbed points.

To shed light on the function of these objects we studied a collection of osseous points from the Dutch North Sea. Since these artefacts were recovered in secondary deposition, we based our interpretation only on wear traces documented on the points. In addition, to create a reference collection of relevant hafting traces, we carried out an experiment to test whether we can identify different hafting designs based on wear trace characteristics and residue distribution patterns. Hafting methods can inform us about the technological and cultural choices of the Mesolithic people of Doggerland. Different hafting designs may have been selected for different hunting activities or because of their efficiency over other methods [21].

Combining these results creates complete biographies of barbed and unbarbed osseous points. This adds new information relevant to the debate about the function of Mesolithic barbed tips and informs us of the technology and hunting strategies of the Doggerland inhabitants during the beginning of the Holocene.

## 2. Materials and methods

### 2.1. Archaeological points

The assemblage of Dutch North Sea osseous points consists of more than 1000 barbed and unbarbed points that have been recovered from several locations in the province of South Holland. These finds come from waterlogged sediments, which predate the final inundation of the North Sea basin around 6000 years ago [22]. The sand is dredged from known locations situated several miles off the coast and used for beach replenishment and construction works [23]. Therefore the points are subsequently found along the beaches. The finds document the Early Holocene occupation of the drowned North Sea prehistoric landscape, named Doggerland [24], which stretched from the Netherlands to Great Britain, Denmark, and Norway [25]. The Doggerland materials is precious for the Netherlands, where most Palaeolithic and Mesolithic sites are buried many meters below the surface. It has been suggested that Doggerland was the heart of the northwestern European Mesolithic and likely holds one of the most comprehensive records of the Holocene [13]. However, since relevant archaeological layers in the sea can be challenging to access, surface finds are the only means of investigating the archaeological heritage in the North Sea. Thanks to the exceptional preservation of organic materials (bone, antler, and adhesive residues) the investigation of barbed points from the Dutch North Sea, despite being surface finds, has the potential to broaden our knowledge of technology and behaviour in Mesolithic Doggerland and provide us a unique window into the inhabitants of wetlands.

Direct ^14^C dates on 15 barbed points from Dutch Doggerland confirmed their attribution to the Mesolithic period, roughly between 9950–7300 years ago [10,26]. The first large-scale study of these objects was conducted by Verhart [27]. More recently, Spithoven analysed a larger assemblage of points with a morphometric and functional approach [28,29]. Verhart distinguished two categories of points based on morphometric attributes: 1) Small points, less than 85 mm in length, were likely used as arrow tips for small prey, fishing, and fowling. 2) Large points, over 94 mm, were likely used as spear tips or harpoons for large marine and terrestrial animals [14,27]. The distinction between these groups has now been set at a length of 88.5 mm [10]. Both authors agreed to classify these tools as projectile tips but did not find enough evidence to identify with confidence the prey hunted.

**Fig 1:**
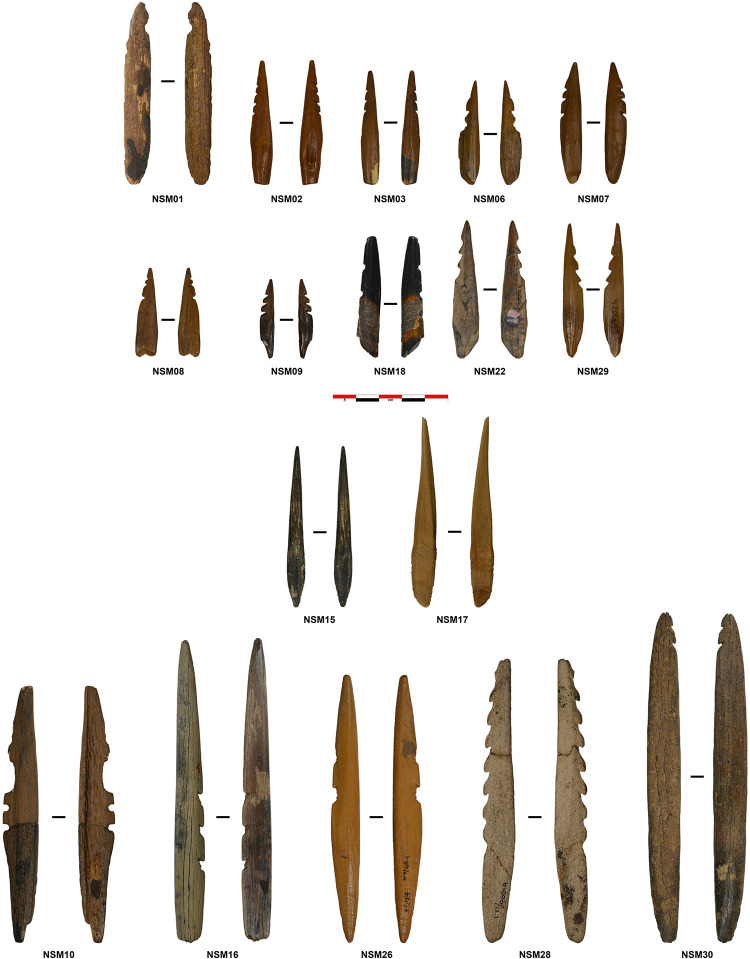
Overview of the archaeological points analysed in the study.

This study consists of 17 osseous points (Fig 1) that we selected because all show macroscopic indications of hafting, such as residues/staining, binding impressions, and a difference in surface morphology and wear between tip and base. We decided to analyse a small sample of tools in great detail, using a wide range of techniques that cannot be applied to large assemblages because then the analysis becomes too time-consuming and too expensive. To reconstruct their use-life, we analysed the objects with a multi-analytical approach integrating morphological, metric, chemical, and spectrographic methods. Destructive analyses were performed only on NSM1, 10, and 30 and on loose residues of NSM18 (Table 1). Regarding the other artifacts, the owners did not provide consensus for destructive sampling. No permits were required for the described study, which complied with all relevant regulations.

**Table 1:** Overview of the archaeological sample of osseous points and destructive and non-destructive techniques applied in this study.

Tool ID	Find location	Raw material	Tool type	Preservation	Weathering	Max length mm	Max width mm	Max thickness mm	Hafting indicator	Analysis
NSM1	Maasvlakte	Bone/ antler	Barbed point	Poor	Cracking, exfoliation	78	11	5	Black residue	3D scan, use-wear, GC-MS, ZooMS, ^14^C
NSM2	Rockanje	Bone	Barbed point	Excellent	Light corrosion	53	9	5	Brownish residue	Use-wear
NSM3	Rockanje	Possibly bone	Barbed point	Good	Modern damage	49	8	4	Black residue	Use-wear
NSM6	Rockanje	Possibly bone	Barbed point	Excellent	No	45	9	5	Binding impressions	3D scan, use-wear
NSM7	Rockanje	Bone	Barbed point	Excellent	Light corrosion	52	9	5	Incisions?	Use-wear
NSM8	Rockanje	Bone	Barbed point	Good	No	38	9	3	Black residue	Use-wear
NSM9	Rockanje	Bone	Barbed point	Good	Corrosion, cracking	34	6	3	Difference in surface preservation	3D scan, use-wear
NSM10	Rockanje	Bone	Barbed point	Moderate	Exfoliation, modern fracture	108	15	6	Black residue	3D scan, use-wear, GC-MS, ZooMS
NSM15	Rockanje	Possibly bone	Unbarbed point	Moderate	Cracking, exfoliation	68	7	4	Black residue	Use-wear
NSM16	Pijnacker	Possibly bone	Barbed point	Good	Cracking, exfoliation	127	13	6	Black residue	3D scan, use-wear
NSM17	Rockanje	Bone	Unbarbed point	Excellent	No	80	9	4	Binding impressions	Use-wear
NSM18	Maasvlakte 2	Bone/ antler	Barbed point	Good/ Excellent	Cracking	51	9	4	Black residue	3D scan, use-wear, GC-MS*, ^14^C*
NSM22	Maasvlakte 2	Bone/ antler	Barbed point	Good	Cracking, exfoliation, modern residue	58	11	5	Black residue	Use-wear
NSM26	Maasvlakte 2	Bone/ antler	Barbed point	Good/ Excellent	Corrosion	113	13	6	Binding impressions	Use-wear
NSM28	Maasvlakte	Bone/ antler	Barbed point	Moderate/ Good	Cracking, Modern fractures	118	13	3	Black residue	Use-wear
NSM29	Maasvlakte	Bone/ antler	Barbed point	Excellent	No	58	9	5	Discolouration	Use-wear
NSM30	Zandmotor	Bone	Barbed point	Moderate	Cracking, exfoliation	138	15	7	Discolouration	3D scan, use-wear, ZooMS

For NSM18, the destructive analyses (GC-MS, ^14^C-AMS), indicated with *, were conducted on loose residues.

**Fig 2:**
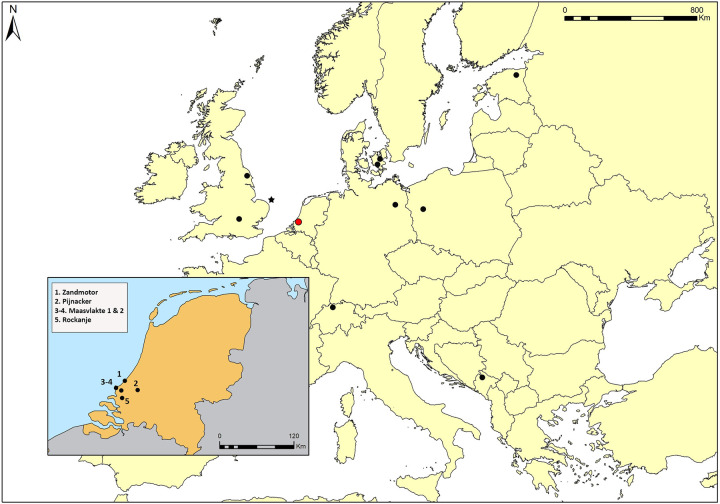
Finding locations of the analysed Dutch North Sea points (in the close-up) and locations of archaeological sites cited throughout the text. From the north: Kunda (Estonia), Ulkestrup and Tåderup (Denmark), Star Carr and High Furlong (England), Friesack (Germany), Krzyż Wielkopolski 7 (Poland), Abri of Liesbergmühle (Switzerland), Odmut (Montenegro). The star represents the finding location of the Colinda point at Leman and Ower Banks.

The points come from several find locations, generally present-day beaches (Fig 2). The points were found along the coast at Rockanje beach (N = 9), the Maasvlakte (N = 6), and the Zandmotor (N = 1). One point was found at Pijnacker, which is located roughly 20 km from the coast, during the construction of a residential area [28]. Most of the points are owned by private collectors except for four (NSM22, 26, 28, 29) that belong to the Rijksmuseum van Oudheden (Leiden, NL).

The state of preservation of the points varies from poor to excellent. This was assessed based on the macroscopic presence of weathering and modern damage. Cracking, corrosion, and exfoliation are the most common natural surface modifications observed on the points, with most showing multiple alterations.

### 2.2 The experimental program

Replicas of Mesolithic bone barbed points were made from deer metapodials (S3 Table 1 in S1 Table). Cutting the blanks and the rough shaping was done with modern tools while the barbs were produced using flint tools. The final shaping was done using flint flakes and a grinding stone (sandstone) to match the production traces observed on the archaeological tools. The experimental bone points were then hafted to fletched pine arrow shafts (length: 830 mm, diameter: 9 mm). Two points were hafted with birch bark tar only. On 16 points tar was used in combination with deer sinew (N = 8) and lime bast (N = 8) bindings. Dried deer sinews, collected from metapodia, were first pounded with a rounded cobble to separate the fibres, moistened with spit, and then wrapped around the point. Raw lime bast fibres, obtained from lime bark stripes, were moistened in water before being used as bindings. Fibres were used plain and not twisted. On 10 points the tar served as bonding material, on eight points it was used for coating lime bast and sinew bindings. The shafts were either bevelled (N = 9) or split (N = 9) (S4 Fig 1 in S1 Fig). In total, we tested six different hafting arrangements and all tests were duplicated (Table 2) [inspired by 14,30].

**Table 2:** Hafting arrangements tested during the experiment.

Hafting type	Shaft	Adhesive	Bindings	Hafting method	Nr of experiments
1a	Split	Birch tar	Sinew	The base of the point (covered in tar) is inserted into the split extremity of the shaft. Bindings are used to secure the point	2
1b	Split	Birch tar	Lime bast	The base of the point (covered in tar) is inserted into the split extremity of the shaft. Bindings are used to secure the point	2
2a	Split	Birch tar	Sinew	The point (without tar) is inserted into the split extremity of the shaft and secured with bindings. The bindings are then coated with tar	2
2b	Split	Birch tar	Lime bast	The point (without tar) is inserted into the split extremity of the shaft and secured with bindings. The bindings are then coated with tar	2
3a	Bevelled	Birch tar	Sinew	The point is hafted with tar on a bevelled shaft and secured with bindings	2
3b	Bevelled	Birch tar	Lime bast	The point is hafted with tar on a bevelled shaft and secured with bindings	2
4a	Bevelled	Birch tar	Sinew	The point is secured on a bevelled shaft with bindings. The bindings are then coated with tar	2
4b	Bevelled	Birch tar	Lime bast	The point is secured on a bevelled shaft with bindings. The bindings are then coated with tar	2
5	Split	Birch tar	-	The base of the point (covered in tar) is inserted into the split extremity of the shaft	2
6	Bevelled	Birch tar	-	The point is hafted with tar on a bevelled shaft	2

The arrows were shot into a ballistic jelly cube covered with leather (2 mm thick) [31,32] using a wooden self-bow mounted on a shooting mechanism (S4 Fig 2 in S1 Fig). The use of a mechanical shooting device has the advantage of reducing variations related to a human archer [33,34]. The distance between the front face of the target and the bow was 2 metres to improve accuracy and reduce variability. A piece of foam was placed behind the target to prevent arrows from getting lost after a missed shot. The arrows were shot with an average speed of 39 m/s, simulating the average speed of a traditional bow [33]. Speed was recorded with a Caldwell ballistic precision chronograph. The arrows were shot a maximum of 25 times to allow hafting traces to develop. When the haft-bond failed, we did not re-haft the projectiles unless the failure was caused by hitting the foam behind or below the target or passing through the target and hitting the ground. The ballistic jelly target was replaced approximately every 18 shots to ensure similar impact conditions and penetration resistance.

### 2.3 Morphometric analysis and 3D

For each archaeological object we recorded the raw material (bone or antler), tool type, maximum length, maximum width, maximum thickness, number of barbs, presence of broken, damaged, and reworked barbs, barb incision shape, base morphology, and base cross-section accordingly to previous classifications [27,29]. The identification of the raw material was based on optical examination of the inner material surface and comparison with bone and antler natural and modified fragments from the reference collection of the Laboratory for Material Cultural Studies (Leiden University, NL).

We created 3D models using close-range photogrammetry to create a permanent record of the points selected for destructive analysis and points with relevant hafting traces (S2). Pictures were taken with a Sony A6300 camera equipped with a 50 mm lens. The points were placed on a hand-operated turntable and manually photographed. The smaller objects (NSM1, 6, 9, 19) were photographed at two different height stages. One image every 5° was captured for each face, totally 72 per whole rotation. The larger objects (NSM10, 16, 30) were photographed at three height stages for each face. For large points, a whole rotation comprised 45 photographs, one every 8°. The images were processed, and high-resolution models were created and properly scaled in Agisoft Metashape 1.6.5.

### 2.4 Use-wear analysis

Macro and microscopic wear traces and residues on the archaeological and experimental samples were analysed using established methodologies [e.g., 35–37]. For the low power examination, we employed a Leica M80 stereomicroscope with an external light source and magnifications ranging from x7.5 to x60 and equipped with a Leica MC120 HD camera. The high power examination was done with a Leica DM6000 M metallurgical microscope fitted with incident light, bright field illumination, polarising filters, and magnifications ranging from x50 to x500. Images were taken with a Leica DFC450 camera. We documented edge-removals, rounding, polish, striations, and residues. We also recorded asymmetry, rough finishing, axis changes, and striations superimposed to production traces as evidence of tool curation and maintenance following the methodology developed for Magdalenian bone points [38,39]. Since the archaeological points have a complex post-depositional story and have been handled and curated, we used the location and distribution of wear traces and residue and their association as fundamental criteria to discern between post-depositional and use evidence [40]. Wear traces were evaluated based on the experiment included in this paper, the experimental reference collection available at the Laboratory for Material Cultural Studies (Leiden University, NL), which comprises more than 4000 experiments including more than 200 bone tools used in varied activities and contact materials, on a reference collection of three unmodified bone fragments recovered from the Zandmotor beach (NL), and previously published literature.

The points were subjected to macrofracture analysis to assess whether they were used as projectiles [41–44]. We analysed fracture types, their position, and distribution patterns, to infer the activity that caused the breakages [45]. Experiments have shown that the only diagnostic impact fractures (DIFs) resulting from the longitudinal impact on bone tools are spin-off fractures larger than 6 mm and bifacial spin-off fractures [41,45]. Burin-like fractures (impact burination), which develop on stone projectiles, are generally very rare in bone points [42]. Other fracture types (bending fracture with step, hinged or feathered termination, snap fracture, and crushing) can be caused either by impact, accidental breakage, or post-depositional processes such as trampling. Since fracture variability is extremely high and fractures could also occur accidentally or after deposition, the combination of the macrofracture method, use-wear and residue analysis is fundamental to identify prehistoric hunting tools.

### 2.5 Dating, GC-MS, and ZooMS

We directly dated two adhesive residues to establish the ages of points 1 and 18. ^14^C-AMS dating was performed at the Centre for Isotope Research at Groningen University (NL). The sample from point 1 was pre-treated with acid only (A) because the material was too vulnerable and too small for the additional base and second acid pre-treatment steps. The sample from point 18 was pre-treated consecutively with acid, a base and a second acid step (ABA-protocol) [see 46]. The base-step was performed at room temperature instead of 80°C since tar/resin can be vulnerable when treated with alkaline solution at higher temperature (S1). The results were calibrated with OxCal v.4.4 [47] using IntCal20 calibration curve [48].

The black residues on points 1, 10, and 18 were sampled for gas chromatography coupled with mass spectrometry (GC-MS). The residue samples were analysed at Inorganic Systems Engineering (ISE) Laboratory (TU Delft, NL). This method allows the identification of materials-specific organic components, or groups of components, that are used as biomarkers to characterize unknown mixtures [49]. A sample of ~10 mg was removed from each object with a sterile scalpel blade. The samples were prepared and analysed by GC-MS following the same methodology employed by Regert et al. [50] and Urem-Kotsou et al. [51] (S1). The GC-MS analyses were performed on an Agilent 7890B gas chromatograph system with a split/splitless inlet, coupled with an Agilent 5977B EI MSD interface, an FID and a splitter with corresponding EPC pressure control to achieve this. The GC was fitted with a nonpolar Agilent J&W DB5 MS column. GC-MS chromatograms are interpreted using National Institute Standard and Technology (NIST). The mass spectra were matched against those of authentic standards (betulin and lupeol), by using previously published data and the NIST library.

Bone samples were collected from points 1, 10, and 30 for zooarchaeology by mass spectrometry (ZooMS) analysis, conducted at the York University BioArCh Laboratory (UK). With this technique unique collagen biomarkers are used to fingerprint and identify species of origin from small amounts of bone. One sample of ~10–20 mg was taken from each point using a sterile metal scalpel blade. The samples were analysed with the ammonium-bicarbonate (AmBic) protocol [52]. The non-destructive buffer extraction was opted because of the small sample sizes (S1). The samples were run on a Bruker ultraflex MALDI-ToF instrument. The resulting mass spectra were interpreted by comparing the peaks to a list of published peptide marker series for all European, Pleistocene medium to large size mammals [53].

## 3. Results

### 3.1. Shape and morphometrics of archaeological points

The sample features 15 unilateral barbed points and two unbarbed points. Seven points have three barbs; points with more than four barbs are rare. Four points have at least one broken barb. Ten points have at least one damaged barb. Five display traces of a reworked barb. The barbs were mainly cut with oblique incisions (type 2; see [27]). Different base morphologies and base cross-sections are visible across the sample without one being predominant (Table 3). Twelve points belong to the group of small points (length <88.5 mm), while five to the group of larger points (length >88.5 mm) [10,27].

**Table 3:** Results of the typological and morphometric analysis of the archaeological points. Barb incisions shapes as define by L. Verhart [27].

		N	%
**Raw material**	Bone	7	41
	Possibly bone	4	24
	Bone/antler	6	35
**Tool type**	Barbed point	15	88
	Unbarbed point	2	12
**N of barbs**	1	2	11
	2	2	12
	3	7	41
	4	2	12
	5	1	6
	8	1	6
	N.A.	2	12
**N broken barbs**	0	11	65
	1	3	17
	2	0	0
	>2	1	6
	N.A.	2	12
**N damaged barbs**	0	5	29
	1	9	53
	2	1	6
	>2	0	0
	N.A.	2	12
**Reworked barb**	0	10	59
	1	5	29
	N.A.	2	12
**Barb incision shape**	Type1. One horizontal incision	2	11
	Type2. One oblique incision	8	47
	Type3. Horizontal parallel incisions	0	0
	Type4. Series of oblique incisions	1	6
	Type5. Two crisscross oblique incisions	1	6
	Type6. Series of crisscross oblique incisions	2	12
	Type7. Incisions like type 5 and 6 where the bottom of the inside angle was widen by cutting away some bone	1	6
	Type8. Incisions like type 5 and 6 where the bottom of the inside angle is widen by parallel horizontal incisions	0	0
	N.A.	2	12
**Base morphology**	Oval	3	18
	Squared	3	18
	V-shape	4	23
	Asymmetrical V-shape	5	29
	N.A.	2	12
**Base cross-section**	Flat	7	41
	Oval	4	24
	D-shape	5	29
	Flat/D-shape	1	6
	**Tot**	**17**	**100**

### 3.2 Ballistic experiment and experimental hafting traces

**Fig 3:**
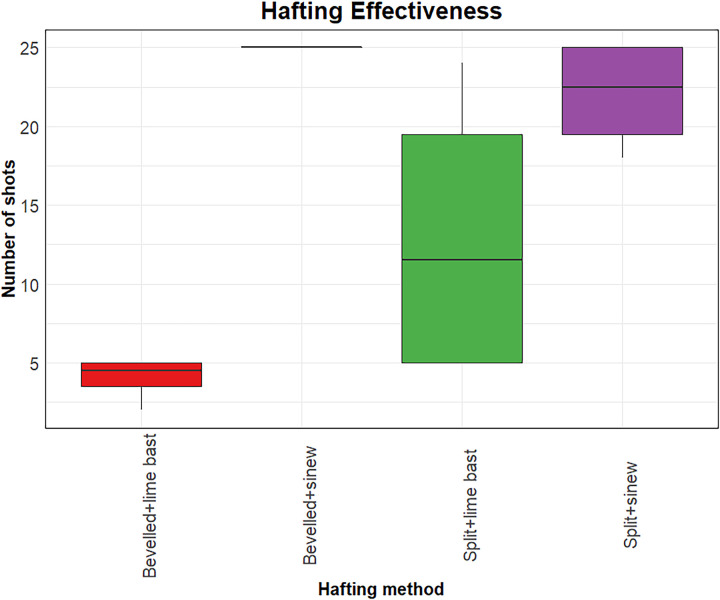
Box and whiskers plot showing the relationship between hafting methods and hafting effectiveness.

Six arrows lasted 25 shots, two of them hafted with the split shaft and four with the bevelled shaft, all of which were secured with sinew bindings (S2 Table 2 in S1 Table). However, overall, the arrows hafted with the split shaft lasted longer than the ones with the bevelled shaft (mean 19.28 vs 14.5). Arrows secured with sinew bindings lasted longer than those fixed with lime bast (mean 23.5 vs 9) (Fig 3). Sinew bindings were more resistant than lime bast ones, broke less frequently and allowed a better fixation of the point. We assessed these results with a non-parametric Mann-Whitney U test in Statistica by StatSoft. There are no significant differences in the performance of split and bevelled shafts (U = 30.50, p = 0.91). However, sinew bindings are significantly more effective than lime bast bindings (U = 5.50, p<0.01). Five arrows were re-hafted because the tips dislodged by accident during firing. The point hafted on the bevelled shaft without bindings dislodged at the first shot. This experiment was repeated with the same result. The point hafted on the split shaft without bindings lasted for 11 shots before the shaft split. This experiment was repeated, and the shaft split after nine shots.

**Fig 4:**
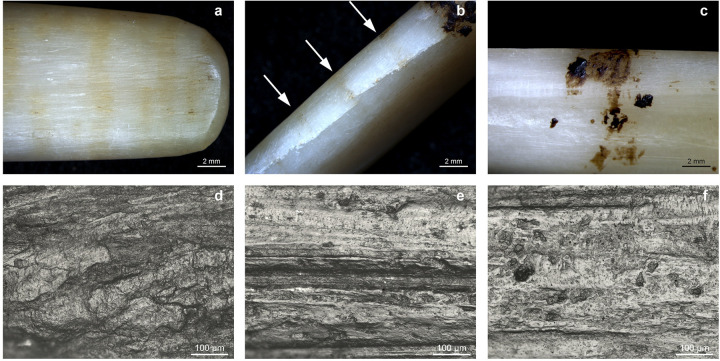
Selection of use-wear traces visible on the experimental bone points. a-b) discolouration in parallel bands due to bindings (7.5x); c) tar residue at the haft limit and tar discolouration. Note the difference in colour between the hafted part and the non-hafted one (7.5x); d) greasy dull polish from sinew bindings (200x); e) smooth and matt polish from lime bast bindings (200x); f) smooth, domed polish on the mesial area probably from contact with the wooden shaft (200x).

Hafting traces developed on 10 of 18 experimental points (Fig 4, S2 Table 2 in S1 Table). Discolouration of the hafted part is visible on six points. On four points, discolouration was caused by the tar, while on two, the discolouration is due to bindings (Fig 4A and 4B). The discolouration caused by bindings is distributed in bands parallel to each other; this pattern was not observed for the tar-stained pieces (Fig 4C). Additionally, binding discolouration affects areas of the tool where tar was absent. Discolouration due to tar is visible on both faces on the points hafted in the split shaft while on one face only for those hafted with the bevelled shaft. Macroscopic binding impressions did not develop on the points even though some of them were left hafted for several weeks. None of the experiments displays macrofractures.

**Fig 5:**
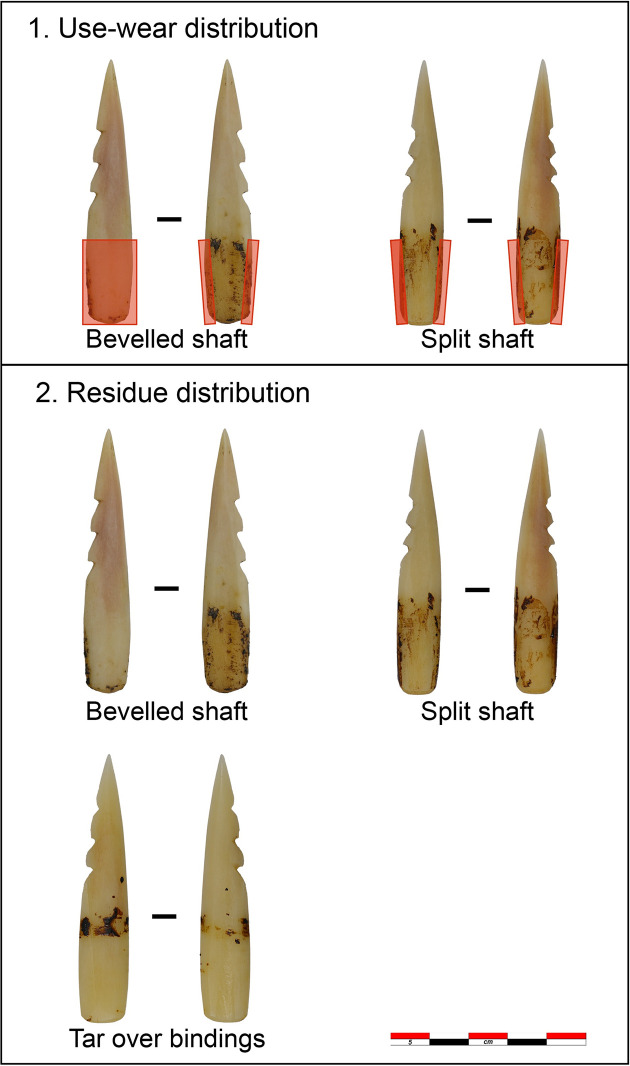
Location and distribution of use-wear traces and residue according to the different hafting methods tested in the experiment.

Nine points show hafting polish. Eight of the nine points with polish display a rough, greasy, and predominately dull polish resulting from the contact with sinew (Fig 4D). One displays a smooth, matt, and bright polish from contact with lime bast (Fig 4E). Polish developed on seven of the eight points bound with sinew, and on one of eight points bound with lime bast. Four points display a transverse directionality in the polish. The polish is limited to the proximal end of the tools. On the points hafted on the bevelled shaft, the polish is visible on one of the flat surfaces of the tool and on the lateral sides. On the points hafted with the split shaft, the polish developed only on the lateral sides (Fig 5). Two points display a smooth, domed, bright polish on the mesial area (Fig 4F) probably resulting from the contact with the wooden shaft. Areas in contact with the adhesive did not develop microwear traces.

Residue distribution varies according to the hafting arrangement employed (Fig 5). The residues are distributed on both faces of the points in the split haft and one face only in the bevelled points. In both cases, residue may also be present on the lateral sides. On points on which the adhesive was used for coating the bindings, the residue and discolouration preserve only at the haft limit.

### 3.3 Use-wear and residue analysis of archaeological points

The examination of the unmodified bone fragments collected at the Zandmotor beach provided a comparison for interpreting post-depositional traces. Post-depositional polish on these bone fragments has a random distribution with no directionality. Some locations of the surfaces display a flat, smooth, and reflective polish with deep long striations.

Modern contaminations visible on the archaeological osseous points consist of ink and clear varnish for labelling, glue for repairing, plasticine, and wood glue. Wood glue, commonly applied to consolidate organic tools after desalting, hindered the microwear analysis resulting in two of 17 examined points being excluded from the use-wear analysis.

#### Macrowear traces

**Fig 6:**
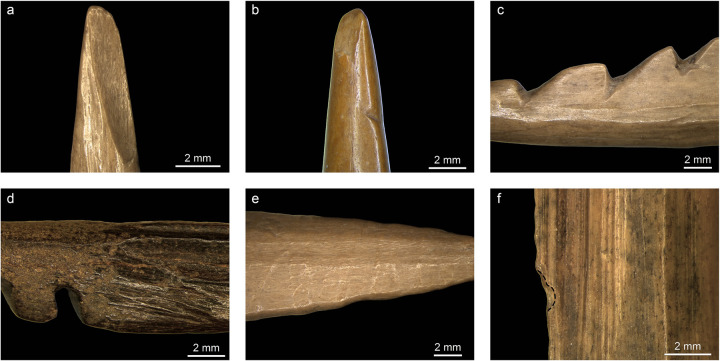
Selection of macrowear traces documented on the archaeological points. a) impact fracture on NSM29 (12x); b) reworked barb on NSM02 and tip fracture (10x); c) difference in barbs shape on NSM29 (7.5x); d) difference in surface preservation between the tip and the base on NSM09 (12.5x); e) binding impression on NSM26 (7.5x); f) edge-rounding and edge-removal caused by bindings on NSM06 (16x).

We documented a total of 24 macrofractures on 15 points, with most showing multiple fractures (Table 4). All of these 15 points present at least one fracture on the tip. Nine of these points also display damage at the base. Crushing is the most visible fracture type (N = 7), followed by bending fracture with step termination (N = 6) and hinge termination (N = 3). Unifacial spin-off fractures are visible on three points, while only one bifacial spin-off fracture is visible. Three points display a snap fracture with a diagonal profile. A single impact burination fracture is observed in the studied assemblage. Only NSM29 displays a diagnostic impact fracture (DIF) [cf. 41]. This point has on the tip a bending fracture longer than 6 mm with step termination (Fig 6A) from which a bifacial spin-off fracture was initiated. This fracture type can hardly occur in another way than through use as a hafted projectile [45, p. 7]. However, considering that osseous barbed points are known to be used as projectiles, it is likely that more of the fractures documented resulted from impact damage; either direct (tip) or recursive (base). Additionally, the fractures visible at the proximal end of the barbed points are consistent with wear from fixed hafting [54].

**Table 4:** Results of use-wear and residue analysis on the archaeological points.

Tool ID	Macrofracture	Barbs macrowear	Use microwear	Hafting microwear	Residue	Observations
NSM1	Tip: fracture but bad preservation	•1 damaged	•Bright and smooth polish domed topography longitudinal directionality	•Isolated spots of bright smooth polish with transverse directionality and striations	•Black residue, mesial proximal part	
NSM2	Tip: bending hinge termination, minor damage	•1 reworked •1 damaged	Not visible	•Left proximal edge: compression due to bindings	•Brownish layer full of cracks on platform	
NSM3	Tip: snap and unifacial spin•off step termination	•1 damaged	Not visible	•Binding impressions on lateral edges •Edge-damage right mesial edges	•Staining base and distal part	•Modern damage on one side
NSM6	Tip: snap	•1 reworked •1 damaged •Difference in shape and size. 2^nd^ barb smaller than 1^st^ one	•‘Corrugate’ polish. Mainly dull rough and greasy, but with smooth matt and bright spots	•Binding impressions on lateral edges •Edge-damage left mesial-proximal edge •Edge-rounding left mesial-proximal edge •Smooth, greasy, bright polish with flat topography and fine transverse striations	•Staining all over the surface •Post-depositional residues all over the surface	
NSM7	Tip: minor damage Base: bending step termination	•1 reworked •1 damaged •Difference in shape and size. 3^rd^ barb is larger and squared compared to the others •Axis change	•‘Corrugate’ polish. Mainly dull rough and greasy, but with smooth matt and bright spots	•Edge-damage right mesial edge •Smooth, greasy, bright polish with flat topography and fine transverse striations	•Staining on barbs •Post-depositional residues	
NSM8	Tip: minor damage Base: crushing with hinge and step terminations	•2 damaged/reworked •Difference in shape and size. 2^nd^ barb bigger and rounded than 1^st^ one •Axis change	•‘Corrugate’ polish. Mainly dull rough and greasy, but with smooth matt and bright spots	•Smooth, greasy, bright polish with flat topography and fine transverse striations	•Staining distal part •Black residues, reddish at extremities, proximal part	
NSM9	Tip: bending step termination Base: bending step termination, minor crushing	•1 damaged	•Not visible, very corroded tip	•Difference in surface preservation. Tip very corroded compared to base •Binding impressions lateral edges •Edge-damage right mesial edge	•No residue	
NSM10	Tip: modern fracture Base: impact burination hinge termination	•3 broken (modern)	N.A.	•Binding impressions lateral edges	•Staining base, barbs, and tip •Clump of residue proximal part. Black.	•Modern wood glue on the surface except on the residue •Modern fracture. Point repaired with glue
NSM15	Tip: minor damage	N.A.	•Fine transverse striations	•Surface very worn and corroded	•Staining all over the surface	
NSM16	Tip: crushing with hinge step terminations, unifacial spin-off step termination Base: bending step termination, minor crushing with hinge step terminations	•2 minor damage	N.A.	Not visible	•Staining proximal and distal •Clump of residue proximal part. Black.	•Wood glue all over the surface except on the residue
NSM17	Tip: bending hinge termination	N.A.	•Smooth and bright polish •Fine transverse striations	• Smooth, greasy, bright polish with flat topography and diagonal and longitudinal striations	•Post-depositional residues all over the surface	
NSM18	Tip: crushing Base: step terminations	•1 chipped	•‘Retrieval’ cut marks	Not visible	•Black residue proximal-mesial part with fibres impressions.	
NSM22	Tip: minor damage Base: hinge termination		•Bright and smooth polish domed topography longitudinal directionality (2^nd^ barb)	•Binding impressions lateral edges •Edge-damage right mesial edge •Post-depositional polish	•Staining proximal-mesial part. Black, compact, homogeneous, very polished with lot of striations •Modern residue (plasticine)	
NSM26	Tip: snap Base: crushing with step termination and unifacial spin-off step termination	•1 reworked	Not visible	•Binding impressions encircling the base •Edge-rounding proximal part	•Grey staining (modern?) •Microscopic residues mostly located on the distal part (more rough). Residues covered with glue/varnish. Post-depositional • A few reddish/orange residues, very granular	•Curation hinder the observation of use-wear and residues. Striations everywhere probably connected to brushing
NSM28	Tip: minor damage	•1 broken •1 chipped •7^th^ 8^th^ barbs very rounded and not pointed compared to others	Not visible		•Staining barbs and distal part •Black residues, reddish at extremities, proximal part •Reddish/brownish residue proximal part with diagonal orientation	•Modern fractures. Point repaired with glue
NSM29	Tip: bending step termination (>6mm) and bifacial spin-off step termination. DIF Base: crushing hinge terminations	•1 reworked •3^rd^ barb sharper compared to the others	Not visible	•Binding impressions lateral edges •Edge-damage right mesial edge •Edge-rounding proximal part •Smooth, matt, metallic polish with flat topography and fine transverse striations •Discolouration proximal part	•Isolate black residues on the platform, inside barbs incisions and tip. Very reflective	•Modern glue/varnish in several locations
NSM30	Indet. Bad preservation	•1 chipped	Not clear	•Binding impressions lateral edges •Discolouration proximal part	•Staining proximal part	

N.A. = not applicable.

Five barbed points display a reworked barb (Table 4). Reworked barbs were mostly partially removed (N = 4), leaving only a slightly raised scar (Fig 6B), or completely removed (N = 1). These scars are always located on the distal part of the points close to the tip and never observed on the mesial or basal area. Those traces may be associated with removed fractured/damaged or blunted barbs [cf. 38] Besides, resharpening and repairing the tip by grinding and scraping would have resulted in a shortening of the point at the tip affecting the barbs at that end which may have been removed to maintain a sharp functional extremity. In addition, we identified traces of rejuvenation of the distal area. Rejuvenation resulted in a visible modification of barb shape and sometimes asymmetry and axis change [38]. Five points show a clear difference in shape or size between the top barb(s) and the lowest ones (Fig 6C). In two cases (NSM7 and 8), a change in the point axis is also visible. This evidence is associated on two points with a rough finishing of the distal area, with coarse striations macroscopically visible and overlaying production traces. These traces strongly suggest that these objects were often repaired and reworked and their use-life extended as much as possible. We also observed the so-called ‘retrieval’ cut marks on the distal-mesial section of NSM18. These marks are described as short, oblique, and isolated incisions that form when the point is retrieved from inside a carcass or cut away from the haft for repair or retooling [38, p. 347].

Two points display surface corrosion at the tip, while their proximal surfaces are better preserved (Fig 6D). Two points show discolouration of the proximal part (Table 4). The differences in surface modification between the proximal and distal parts demonstrate that these parts of the points were exposed to different environments. Ten points have macroscopic binding impressions (Table 4). Binding impressions appear as regularly spaced depressions on the bone surface (Fig 6E). Nine points have binding impressions on the lateral sides only, whereas one point (NSM26) has binding impressions encircling the base. These impressions are associated with edge-damage on six objects and with edge-rounding on three (Fig 6F).

#### Microwear traces

**Fig 7:**
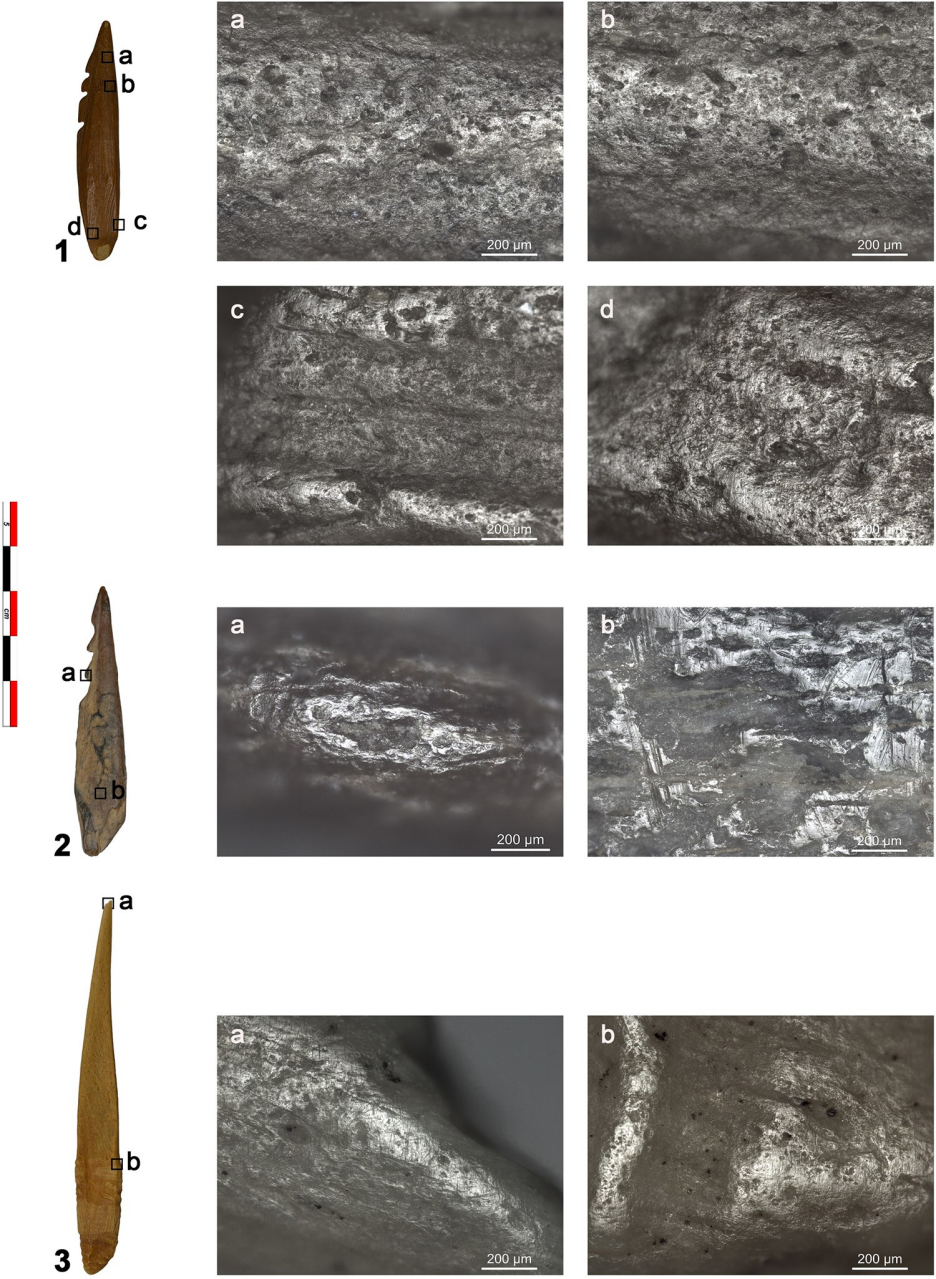
Selection of microwear traces documented on the archaeological points. 1. NSM07; a-b) polish with corrugated texture likely resulting from contact with fish; c-d) polish and fine transverse striations from sinew bindings. 2. NSM22; a) bright smooth polish with longitudinal directionality likely resulting from contact with bone; b) post-depositional polish with long deep striations. 3. NSM17; a) polish and transverse striations from boring animal hide; b) smooth and bright polish from hafting. Magnifications 100x.

**Fig 8:**
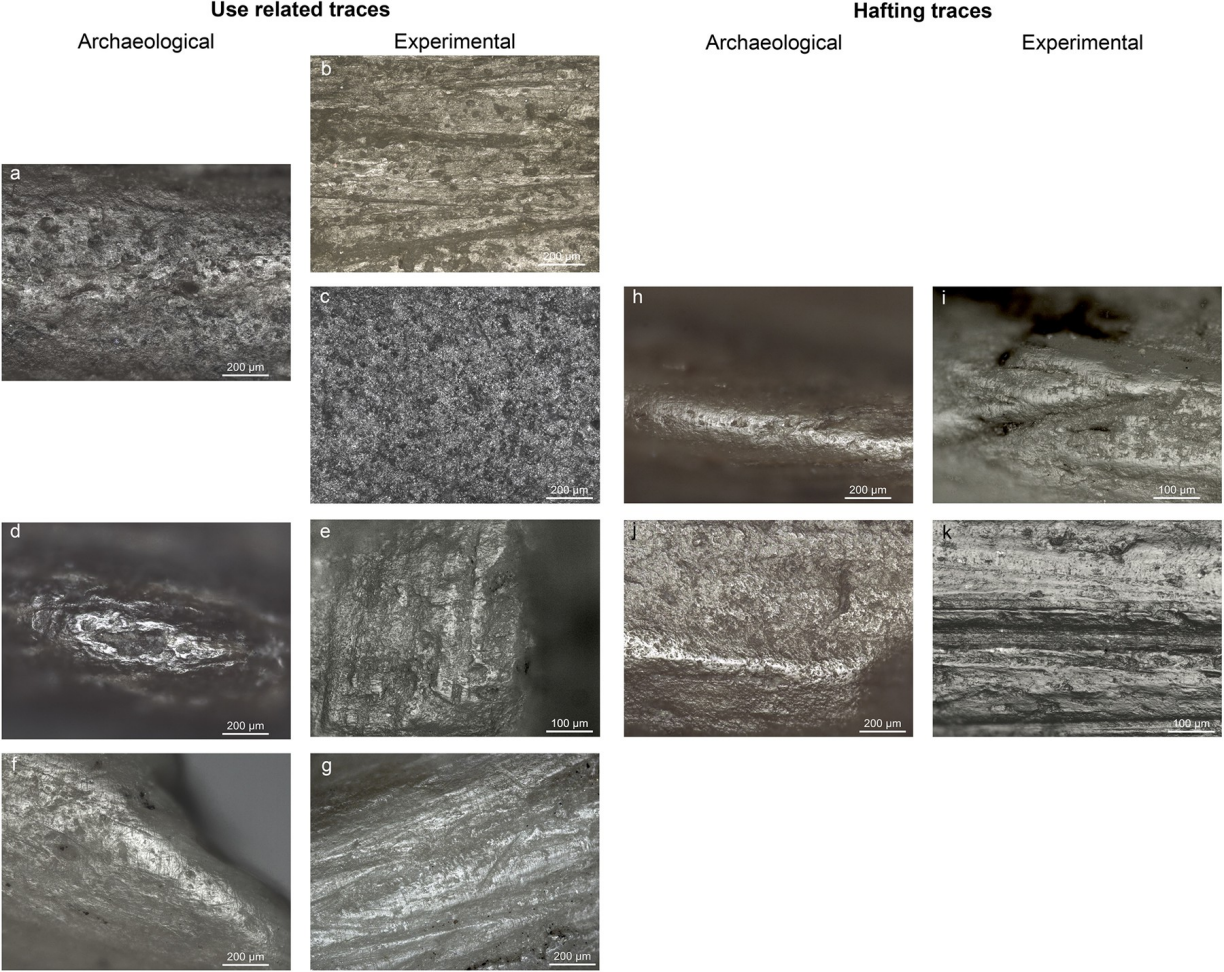
Comparison between archaeological and experimental wear traces. a) fish polish on NSM07 (100x); b) polish on an experimental bone point from shooting salmon (100x); c) polish on an experimental flint tool used to process fish (red snapper) d) bone polish on the second barb of NSM22 (100x); e) bone polish on an experimental point used to shot a carcass (100x); f) polish and short transverse striations on NSM17 from boring animal skin (100x); g) polish and short transverse striations on an experimental borer used to perforate deer skin (100x); h) polish and short transverse striations on the base of NSM08 from sinew bindings (100x); i) polish and short transverse striations from sinew bindings (200x); j) smooth bright polish on the base of NSM29 from plant bindings (100x); k) flat polish from lime bast bindings (200x).

We documented microwear traces related to use on five small barbed points (Fig 7, Table 4). None of the large barbed points display distinctive microwear traces. On three points (NSM6, 7, 8), a ‘corrugated’ polish is visible on the active part. The polish is mainly dull, rough and greasy, but with smooth, matt, and bright spots (Fig 7.1 a-b). Based on the characteristics of the polish (location, distribution, texture, and topography), extensive visual comparisons with the experiment reference collection in Leiden -which includes two bone points shot into a salmon and 48 flint tools used on fish- and existing literature, we interpret these traces of wear to result from contact with fish [55–57]. This polish closely resembles the polish on an experimental point shot into a salmon (Fig 8A and 8B) and on experimental flint tools used to process fish (Fig 8). Polish from contact with fish displays on both flint and bone features of contact with soft and hard materials and it is characterized by a corrugated texture and a dull greasy polish with smooth and bright spots. Two points have a bright smooth polish with domed topography and clear longitudinal directionality. Based on its characteristics, this polish is interpreted as associated with contact with bone and it probably resulted from contact with animal bones during impact. It is located close to the tip of NSM1 and on the second barb of NSM22 (Fig 7.2, a). A similar bone polish is also visible on an experimental point used to shoot a carcass (Fig 8D and 8E). Besides, polish directionality, longitudinal on both experimental and archaeological tools, corroborates the interpretation of the use motion (shooting). Therefore, the use wear traces on barbed tips (NSM1, 6, 7, 8, 22) most closely corresponds to the use wear traces on experimental tools used to hunt fish and land animals.

Two unbarbed points (NSM15, 17) display striations with a transverse orientation, indicative of a boring motion, on the tip. On NSM17, striations are associated with a smooth, greasy, flat, bright polish (Fig 7.3a). Based on the wear traces and comparison between archaeological and experimental microwear, the unbarbed points were likely used as perforators to work animal materials, likely hide (Fig 8F and 8G). At least one of the two unbarbed points bears evidence of hafting. The polish visible at the base resembles the one at the tip (Fig 7.3b). Thus, it is likely that a strip of hide or leather was wrapped around the tool to provide a better grip [21].

Hafting traces were documented on five barbed points (Table 4). Four points (NSM1, 6, 7, 8) display a smooth, greasy, flat, and bright polish on the mesial-proximal area. This polish is mostly visible on the lateral sides of the tools and is better developed on the high reliefs of the surface compared to low areas (Fig 7.1 c-d). On the base of NSM29, a smooth, matt, flat, almost metallic polish is present. Fine, short, transverse striations are always associated with these micro-polishes. These traces are interpreted as originating from sinew and vegetal bindings of the hafting arrangement. Sinew is identified due to the similarities between the archaeological and experimental traces (Fig 8H and 8I). The experimental polish from lime bast does not provide an accurate match for the archaeological material (Fig 8J and 8K) but we can still interpret some of the binding polish as being related to contact with plant material. NSM22 displays a very flat, smooth, and reflective polish with long deep striations on the mesial-proximal area that we interpret as post-depositional (Fig 7.2b).

#### Residue analysis

Residues are present on 12 out of 17 points. Four points (NSM3, 15, 22, 30) display only black staining/discolouration, meaning no physical three-dimensional residues are preserved. NSM9 has no residue. We excluded NSM26 because the residues are located under a layer of wood glue.

Three points (NMS6, 7, 17) have microscopic residues randomly distributed on micro-cracks and grooves of the bone. They are elongated like the cracks, black in colour, and highly reflective when examined with the metallographic microscope in normal light. They are interpreted as post-depositional, most likely related to rooting.

NSM2 displays a brownish residue which is limited to the platform. The residue appears as a homogeneous, smooth, and reflective layer full of cracks. Cracks are not visible in other locations of the point. SEM-EDS analysis confirmed the inorganic nature of the residue. All the EDS spectra show a strong contribution of calcium (Ca) and phosphorus (P), probably originating from the underlying bone’s hydroxyapatite (Ca5(PO4)3(OH)) [58].

Four points (NSM1, 10, 16, 18) display large, visible, black residue on the proximal/mesial portion. These residues are preserved on one side of NSM1, NSM10, and NSM16, and on both sides of NSM18. The residues have a three-dimensional rounded shape, a granular texture, and are sometimes cracked. When examined with the metallographic microscope, the residues are black, brownish at the limits, reflective in normal light, and dull in cross-polarised light. On NSM10 and NSM16, the residues are located at the base of the points. On NSM1, the residue extends 47 mm from the base toward the tip of the object. On NSM18, the residue covers almost half of the point (maximum length 25 mm), covering the third and fourth barbs (Fig 9.1). The surface of this residue displays elongated white/grey striations with a diagonal orientation that may be the remains of fibres or their impressions (Fig 9.1 a-b). However, when analysed with high magnifications (200x-300x), they lack any visible structure, e.g., elongated cells organised in fibrous bundles. Thus, they are likely fibre impressions. The residue on this object is black, terraced, and granular, with some orange inclusions that are semi-translucent in cross-polarised light (Fig 9.1c). On top of the residue, a granular rusty orange layer is visible, likely the result of the degradation of organic material (Fig 9.1d). Based on their distribution, morphology, surface characteristics, and the reference collection, these residues are interpreted as organic adhesive remains.

**Fig 9:**
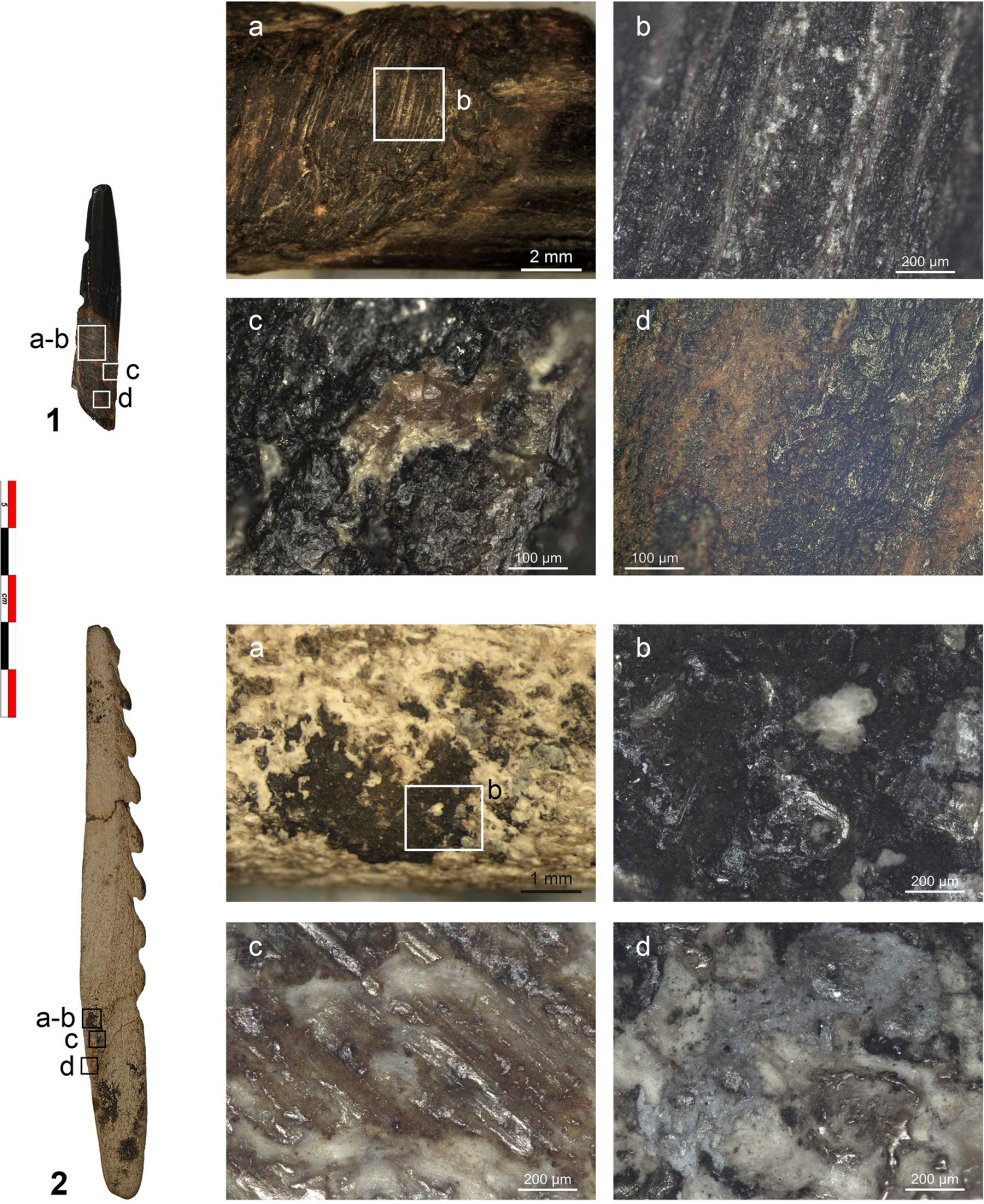
Adhesive residues documented on the archaeological points. 1. NSM18; a) black residue (10x); b) close-up of the possible fibre impressions (100x); c) detail of the orange semi-translucent inclusion (200x); d) granular rusty orange layer on top of the residue (200x). 2. NSM28; a) granular black residue (20x); b) close-up of a (100x); c) granular brownish residue with oblique orientation (200x); d) modern grey residue (200x).

Residues on NSM8 and NSM28 are also interpreted as potential adhesive remains. Micro-residues are visible on both sides of NSM8 towards the base. Bigger residues are black, some are brownish at the limits, with a three-dimensional rounded shape and a granular texture. NMS28 displays a combination of residues (Fig 9.2). Spots of granular black residue are visible at the base, partially covered by a modern grey residue (Fig 9.2a-b-d). Micro-Raman and micro-FTIR indicated the organic nature of these residues. The detected peaks can be assigned to pine tar, pine resin, or birch tar [58]. In addition, on the lateral side of the point, a granular brownish residue with an oblique orientation is visible (Fig 9.2c). The latter is located very close to a modern fracture that was repaired with glue. Therefore, its modern origin cannot be ruled out completely.

### 3.4 Destructive analyses: Dating, GC-MS, and ZooMS

Two ^14^C-AMS dates were obtained from the residue samples belonging to points 1 and 18. NSM1 (GrM-27499) has an estimated age of 9275 years BP and NSM18 (GrM-27889) of 11065 years BP. Calibrated dates range between 10573–10298 cal. BP for NSM1, and 13095–12843 cal. BP for NSM18 (S2). These ages confirm the attribution of NSM1 to the Mesolithic, while NSM18 is attributed to the Upper Palaeolithic (Table 5).

**Table 5:** Results of the destructive analyses on the archaeological barbed points.

Tool ID	^14^C age (yrBP)	Calibrated age range (yrBP)	ZooMS identification	GC-MS
NSM1	9275±35	10573–10298	*Cervus elaphus*	Birch tar
NSM10	N.A.	N.A.	*Bison*—*Bos primigenius*	Birch tar
NSM18	11065±50	13095–12843	N.A.	Possibly birch tar
NSM30	X	X	*Cervus elaphus*	N.A.

X = no result; N.A. = not applicable. ^14^C ages (in yrBP) are calibrated to calendar years with software program: OxCal, version 4.4 *[47]* using calibration curve IntCal20 *[48]*. * age range for 95,4% probability.

The GC-MS analysis of the residues indicates the presence of pentacyclic triterpenoids with a lupane skeleton and their degraded derivates, and saturated and unsaturated fatty acids and diacids (Table 6). These markers are typical of birch bark tar [59,60]. Lupeol and/or betulin are present in two adhesive samples (NSM1 and NSM10), while in NSM18 only degraded products of these compounds are identified. Although degraded, residue samples from NSM1 and NSM10 can be confidently interpreted as birch bark tar. The peaks in the chromatogram of sample NSM18 are low in intensity which makes it hard to identify the chemical components. Some peaks provide a very low match with compounds with betulin structure. Probably, there are remnants of birch bark tar, but the sample is too degraded to allow a confident interpretation based only on GC-MS results. Degradation can be induced by the natural ageing of the material during the burial period or as a consequence of intentional transformation involving heating processes. Since the bone points come from different primary sites, it is possible that NSM18 was subjected to different taphonomic processes compared to NSM1 and NSM10 which influenced the preservation of biomarkers. Besides this, differences in the Palaeolithic and Mesolithic tar production methods can also be responsible for the dissimilarity in the results. It is possible that the adhesive for NSM18 was produced with a different technique or by using higher temperatures, which affected the preservation of molecules. No chemical compounds for other typical adhesive materials, such as pine resin, waxes, or gum are present. It is therefore likely that birch bark tar was used as a single-component adhesive for hafting the points.

**Table 6:** Chemical compounds identified with GC-MS on each sample.

Chemical component	Retention Time	Tool ID		
		NSM1	NSM10	NSM18
Glycerol, 3TMS derivative	11.7	Y	Y	Y
Nonanoic acid, TMS derivative	13.0	Y	Y	Y
Palmitic Acid, TMS derivative	27.0	Y	Y	Y
Bisphenol A, 2TMS	27.3	Y	Y	N
Cyclic octaatomic sulfur	27.2	N	N	Y
Stearic acid, TMS derivative	31.3	N	Y	N
13-Docosenoic acid (E)/ Euricic Acid	34.1	N	N	Y
13-Docosenoic acid, (Z)-, TMS derivative	38.9	Y	Y	N
α-Lupane	43.7	Y	Y	Y
α-Lupane	44.8	N	N	Y
Lupa-2,20(29)-diene	46.0	Y	Y	N
α-Betulin I, TMS	46.4	Y	Y	N
α-Allobetulin	47.2	Y	Y	N
Allobetul-2-ene	49.5	Y	N	N
Lupeol, trimethylsilyl ether	51.0	Y	Y	N
Betulone, TMS derivative	52.8	Y	Y	N
Betulin, bis-TMS	53.2	Y	Y	N
Betulinic acid, O,O-bis-TMS	53.5	Y	Y	N
Allobetulin, TMS derivative	54.2	Y	N	N

Y stands for yes; N stands for no.

Since osseous points are heavily modified by manufacturing, use, reuse, and post-depositional alterations, it is not always possible to identify the raw material. Based on macroscopic observation, seven points are bone, four are probably bone, and six are either bone or antler. Based on the collagen peptide markers NSM1 and NSM30 are identified as Bovidae/Cervidae and NSM10 as cattle (Table 5). For the Mesolithic North Sea area, the label Bovidae/Cervidae refers to a group of species that all share the same markers and consists of either red deer or elk [see 26 and references therein]. In addition, the presence of a peak at m/z 2216 in both samples also suggests we can further specify this to red deer [6]. NSM10 is identified as cattle, which includes bison (*Bison)*, aurochs (*Bos primigenius*), and yak (*Bos grunniens*). The geographical location and age narrowed this down to either bison or aurochs [61]. Even though exceptions are possible, such as three Danish brown bear points [6] and two Doggerland human bone points [26], blanks for bone tools were generally derived from herbivores hunted and brought to the sites [62].

## 4. Discussions

### 4.1 Comparisons

The two barbed points dated with ^14^C-AMS yielded Early Mesolithic and Upper Palaeolithic dates. The Mesolithic age of NSM1 falls in the range of other dates available for Doggerland points (~10000–7000 BP) [26], while NSM18 is the oldest barbed point from the Dutch coast with an age of approximately 13000 years. Only one other barbed point from Doggerland, off the coast of Great Britain, the Colinda point, dates from the Late Palaeolithic. Direct AMS dating of this specimen yielded an age of 13500 cal. BP [63].

The Upper Palaeolithic point in our sample matches the Magdalenian barbed points of the Iberian Mediterranean. Compared to contemporaneous French and Cantabrian barbed tips, Mediterranean ones usually have a single row of small barbs that do not protrude much from the shaft, although a certain degree of variability within the assemblages is visible [64]. The same features characterise both our Upper Palaeolithic and Mesolithic points.

The Mesolithic points of Doggerland compare well in terms of overall morphology, size, and shape of the barbs, with other western European assemblages (Maglemosian tradition) of similar age [e.g., Star Carr, 13, Friesack, 65], except for their reduced length. Despite the high internal variability, which characterises all bone point assemblages, these points show a preference for a unilateral row of small barbs, the absence of distinct bases, and very simple or absent decorations. Base incisions, with an aesthetic and/or functional meaning, are documented on barbed points from Star Carr [11,66] and the Colinda point [67] but are absent in the Doggerland assemblage. Bilateral barbed points, which are represented by one fragmented specimen in the Doggerland assemblage [27], have a more north-eastern European distribution and they likely have roots in the French and Cantabrian Magdalenian tradition [9,68]. The Doggerland points fit well in a unilateral tradition of bone points that has its roots in some of the technocomplexes of the final Upper Palaeolithic.

### 4.2 The function of Dutch Mesolithic osseous points

Our results suggest that barbed and unbarbed points were different tool types with different functions. Use-wear traces indicate that barbed points (N = 15) likely served as hunting weapons. Unbarbed points (N = 2) were used to perforate animal hide. However, a study of a bigger sample is needed to check if this conclusion fits all unbarbed points, or if their function was more diverse.

Some of the studied small barbed points bear traces of contact with mammal bone and others with soft fish tissue, but the size of the prey is unknown. The use of bone points on small mammals like beavers and otters is reported in numerous historical and ethnographic sources [5,19,69]. Direct archaeological evidence for the use of barbed points to hunt beavers comes from the Middle Neolithic layer of Sakhtysh 1, Central Russia, where a fragment of an osseous point was found stuck in a beaver skull [17]. The direct association of barbed points with elk remains confirms their use for hunting large size ungulates as well [13,18]. Ethnographic evidence certifies the use of barbed points for fishing [19] while, for archaeology, this link was often suggested based on indirect associations of osseous points and fish bones [e.g., 15,16]. The microwear traces presented here provide clues supporting the theory of small barbed points being fishing gear as previously proposed [14].

These results reinforce the data on Doggerland environment and the presumed diet of Mesolithic human groups who inhabited this area. At the beginning of the Holocene, the southern North Sea was a rich and diverse landscape characterised by a forested environment with interspersed lakes and marshes [22,23]. This environment provided a wide range of food resources including medium to large ungulates (e.g., red deer, roe deer, aurochs, elk, wild boars), beaver, otter, fish, shellfish, and birds [61,70,71]. Stable isotope analysis of Mesolithic skeletal remains from Doggerland showed a significant freshwater component of their diet, highlighting the importance of aquatic resources [72]. Barbed points were an important part of the hunting equipment and probably complemented the fishing toolkit alongside hooks. Other methods, such as nets and fish traps, which would have yielded a greater number of fish, were likely employed as well. Fish traps from the Netherlands date to the Neolithic [73], but Mesolithic examples of fish traps, nets, and sinkers are well documented in northern and eastern Europe [17,74]. From around 7000 years BP, due to the rapid sea level increase, the Doggerland area transformed from an inland to a semi-marine and then a fully marine environment [75]. Isotope analysis showed that marine resources were also exploited by later Mesolithic hunter-gatherers, although less intensively [72]. A different environment with different prey may have required different hunting tools.

### 4.3 Points size and prey targeted

Both large and small barbed points studied here show macroscopic evidence of their use as projectiles. However, the investigation of micro-polishes on large points in our sample was inconclusive. Therefore, it is still unclear if larger points were designed for different hunting activities or specific prey. More use-wear analysis and dating of points may help to identify different specific functions of barbed points or document a change in their function through time connected to changes in the environment. Such studies are underway in the project Resurfacing Doggerland by Dr. Hans Peeters (NWO AIB.19.009).

Previously it has been suggested that small points were arrow tips and the larger ones were spear tips based on size, weight, and shape of the barbs [14,65]. In addition, the small points would have been used on small prey and the larger tips on larger sized prey. Both ideas can be contested. Before making a connection between the size of the point and the prey, it would be necessary to conduct a functional study to separate proper projectile points from pointed bone tools used in activities other than hunting. As our analysis has demonstrated not all the pointed tools are projectiles [cf. 76], although they are often grouped under this label [e.g., 10,65]. Also, since barbed points are often reworked, the morphology of the barbs we see may result from the practical constraints of resharpening and repairing the object, such as the size or shape of the blank.

If we accept that small barbed points from the Dutch North Sea are unique in terms of their size among the European scenario [10], then we cannot assess their function based on comparisons with other assemblages which are predominantly featuring large points. Many European bone point assemblages are found with *ichthyofauna*, *often pike*, *but their association is not proven*. Because these assemblages do not contain small points, we cannot deduce if there are prey differences based on the point type. Besides, direct evidence of large barbed points associated with large mammal bones, like the example of elk from High Furlong, is too scarce to suggest a strong connection between different sized points and the size of the prey targeted. Furthermore, ethnographic examples highlight the variability of shapes and sizes of points used as arrowheads and spearheads [e.g., 77,78]. Therefore, a typological and functional interpretation based only on the size of the points is not reliable [cf. 1,5,20].

### 4.4 Reconstruction of hafting methods

The location and characteristics of wear traces and residues, together with the experimental results and chemical analysis, provide clues as to how the barbed points studied here were hafted. The tools were attached to their shafts with birch tar and animal and vegetal bindings. The location of the residue on some points on one side of the tool indicates the use of a bevelled shaft, while residues on both sides indicate a split shaft. The location of binding impressions on both lateral edges may point toward either a split or bevelled shaft. There is no clear indication of the preference for the bevelled shaft over the split one. An exception is NSM26, which displays binding traces encircling the base. According to Verhart [27, p. 183], this point may have been entwined to create a better fit into the shaft. We hypothesise that this point was reused and re-hafted several times on a bevelled shaft allowing traces to form on both sides. Moreover, based on the location of binding impressions on the meso-proximal area of the tools, we conclude that these points were not detachable. Impressions left from a harpoon line would have been limited to the mesial part of the implements [cf. 16]. We identified no ’classical’ harpoon points with a detachable head and a line [category A in 1]. None of the analysed implements display features for fastening a line, such as the presence of a basal perforation (linehole), lateral spurs at the base, or the binding barb. In the total North Sea assemblage (N = ⁓1000) only two points classify as possible harpoons [10]. Both are large points and display a single or double notch at the proximal base where the line may have been attached [27, Fig 8 MS133 and Fig 9 KF41]. However, these points may have been firmly attached to a foreshaft and therefore still be part of a composite detachable weapon system. North American Shuawps’ beaver harpoons, for instance, are composed of an osseous barbed point attached to a wooden foreshaft [79]. The morphology of these harpoon points, however, does not differ from other fixed points [1]. Therefore, it is almost impossible to identify their detachable nature if recovered without the shafts.

Micro-polishes on the meso-proximal area of some of the studied tools provide evidence of the binding materials used to secure the points. In some cases, sinew is identified while some of the other binding polishes are plant related. Although our experiments indicate that sinew is a stronger binding material and led to a lower failure rate during the shooting experiments, vegetal fibres are well documented in archaeology [e.g., 11,65,80]. We also cannot completely rule out that the failure of the hafting bond was intentional. Failure would have allowed the point to detach upon impact and rankle in the wound causing more internal damage [cf. 77].

Birch tar was used as a single-component adhesive. The use of birch tar as an adhesive for tools used for fishing is not surprising considering its material properties. Birch tar is not water-soluble and can be reheated and reused many times with almost no detrimental effects on its performance [81]. Evidence of pure birch bark tar adhesive or a mixture of birch tar and pine resins is well-known in the European Palaeolithic and Mesolithic respectively [see 82 and references therein]. However, the majority of points studied here did not have adhesive residues. Although this may be due to a preservation bias, it is conceivable that hafting methods of barbed points did not always necessitate adhesives. Direct evidence from the Mesolithic sites of Friesack (Germany) [65] and Ulkestrup (Denmark) [83] shows that barbed points were not always mounted with adhesives. At Friesack, some points were bound to the shaft with strips of bast without glue. Others were hafted with tar and a combination of tar and bindings [65]. In addition to this, in many examples, indigenous peoples of North America do not use adhesives to bond the points to the shafts but only sinew [77]. This evidence may explain the low number of adhesive residues compared to the relatively high occurrence of binding traces on the North Sea points. Another possible explanation is that North Sea points were mounted with bindings and tar used only as a coating agent as seen, for instance, at Friesack [65] and Krzyż Wielkopolski 7, Poland [84]. Our experiments show that minimal residues preserve on the points when this arrangement is used. Moreover, when used as fishing gear, the bindings likely required some adhesive or sealant to waterproof them.

### 4.5 The long life of barbed points: Reuse, rejuvenation, re-hafting

Reuse and rejuvenation of barbed points seem a common technological behaviours. Our sample of osseous points shows traces of maintenance (rejuvenation, reuse, and reworking). Other studies also documented a large number of rejuvenated and reworked Dutch points [27,29]. Besides rejuvenated tips and barbs, the Doggerland assemblage features fragments of large points that broke and were roughly refurbished into an equivalent tool. The old barbs were ground away, leaving visible scars on the side, while new barbs were cut near the tip [Fig 1, NSM8; 27, Fig 14 KF69]. Maintenance traces are common on other contemporaneous Upper Palaeolithic and Mesolithic barbed osseous points as well [39]. Experimental work [30,62] showed that bone points are more durable and are easier to repair when dull or broken compared to their lithic counterparts.

This evidence highlights that during their use-life, Doggerland barbed points were intensively curated, reused, and re-hafted many times before being discarded or lost. The bone material accommodated this intense reuse, but material selection, e.g., human and brown bear bones [26], may also suggest that these points were imbued with specific cultural and symbolic connotations. The points may also have had special meaning and were therefore used for a very long time by their Mesolithic owners.

## 5. Conclusions

We presented the results of a detailed functional analysis on a sample of 17 barbed and unbarbed points recovered from the beaches of the Dutch North Sea. We reconstructed their use-lives and assessed the animals hunted.

Our sample features the oldest barbed point recovered from the Dutch North Sea, roughly 13000 years old. The other point dates to the Early Mesolithic, roughly 10500 years ago. Morphological similarities, dominance of unilateral barbs, small teeth, and general lack of decoration, between Doggerland and European bone points of the Upper Palaeolithic and Mesolithic periods indicate that these tools were part of a shared European tradition and a systematic component of the hunting kit since at least the end of the Palaeolithic. Macrofractures visible on the tips and bases of barbed points indicate their use as projectiles. We also show that on some small points, the polish closely resembles experimental fish polish, and on others, the polish resembles (mammal) bone polish. We suggest that barbed points were used for hunting both aquatic and terrestrial animals. Prey targeted may have included freshwater fish, ungulates, and animals hunted for fur, such as beavers or otters. Evidence of rejuvenation of small and large points, reuse of large barbed point fragments, and re-hafting underline that these were highly curated tools, and their use-life was extended as long as possible. The bone facilitates extensive reuse, and perhaps the use-lives of the points were extended because they bore a special meaning to the owners. Conversely, unbarbed points do not display impact fractures. The presence at the tip of striations oriented transversely to the axis of the tool indicates their use in boring/piercing activities, likely to perforate hides.

The characteristics of macro and micro hafting traces and chemical analysis help to reconstruct the hafting methods of bone points. Split and bevelled systems were used in combination with birch tar adhesive and sinew and vegetal bindings. Our experiment showed that sinew binding works better than vegetal ones and with tar can create an excellent joint. Considering the large number of points with binding traces and no adhesive residues, bindings may also have been used alone to secure hafts.

Our results highlight that barbed points were dynamic tools that transformed during their life through use, repair, and reuse. The situation may also be similar for unbarbed points. Such transformations may have led large points to become small ones, possibly with a consequent change in their function. Analogies show that both small and large points served multiple different purposes and were used on various prey. Therefore, a functional distinction of barbed weapons between arrow and spear tips based only on morphometrics is not sufficient to account for the complexity and variability of archaeological assemblages. Only by combining a functional approach encompassing use-wear analysis, ethnographic analogies, and problem-oriented experiments, can we concretely demonstrate the precise function of barbed osseous projectile points.

## Supporting information

S1 Fig Figs 1 and 2. (DOCX)

S1 Table Tables 1 and 2. (DOCX)

S1 File Destructive Analysis–Expanded Methodology: ^14^C-AMS dating, Gas chromatography–mass spectrometry (GC-MS), Zooarchaeology by mass spectrometry (ZooMS). (DOCX)

S2 File Extended results: 3D models of bone points with mastic and hafting traces, ^14^C-AMS dating. (DOCX)
