# Correction: The dynamic lives of osseous points from Late Palaeolithic/Early Mesolithic Doggerland: A detailed functional study of barbed and unbarbed points from the Dutch North Sea

**DOI:** 10.1371/journal.pone.0319341

**Published:** 2025-02-11

**Authors:** Alessandro Aleo, Paul R. B. Kozowyk, Liliana I. Baron, Annelou van Gijn, Geeske H. J. Langejans

There are errors in the article title. The correct title is: A functional study of hafted barbed and unbarbed Late Palaeolithic/Early Mesolithic points from the Dutch North Sea. The correct citation is: Aleo A, Kozowyk PRB, Baron LI, van Gijn A, Langejans GHJ (2023) A functional study of hafted barbed and unbarbed Late Palaeolithic/Early Mesolithic points from the Dutch North Sea. PLoS ONE 18(8): e0288629. https://doi.org/10.1371/journal.pone.0288629.

In the Archaeological points subsection of the Materials and methods, there is a sentence missing between the first and second sentences of the third paragraph. The missing sentence is: Seven of these points (NSM2, 3, 6, 7, 8, 9, 18) were previously analysed with a macro and morphometric approach by Spithoven [29] but were reanalysed here to obtain a very high level of detail that is consistent with this study.

In the Microwear traces subsection of the Results, there is an error in the fifth sentence of the first paragraph. The correct sentence is: This polish closely resembles the polish on an experimental point shot into a salmon (Fig 8A and 8B, experiments conducted by Spithoven, [29]).

In the Microwear traces subsection of the Results, there is an error in the tenth sentence of the first paragraph. The correct sentence is: A similar bone polish is also visible on an experimental point used to shoot a carcass (Fig 8D and 8E, experiment conducted by Spithoven, [29]).

In the Points size and prey targeted subsection of the Discussions, there is an error in the first sentence of the first paragraph. The correct sentence is: Both large and small barbed points studied here show macroscopic evidence of their use as projectiles (see also [29]).

In the Points size and prey targeted subsection of the Discussions, there is an error in the first sentence of the second paragraph. The correct sentence is: Previously it has been suggested that small points were arrow tips and the larger ones were spear tips based on size, weight, and shape of the barbs [14, 29, 65].

In the Reconstruction of hafting methods subsection of the Discussions, there is an error in the eighth sentence of the first paragraph. The correct sentence is: We hypothesise that this point was reused and re-hafted several times on a bevelled shaft allowing traces to form on both sides, underlining a hypothesis also made by Spithoven [29].

In The long life of barbed points: Reuse, rejuvenation, re-hafting subsection of the Discussions, there are errors in the first and second sentences of the second paragraph. The correct sentences are: This evidence highlights that during their use-life, Doggerland barbed points were intensively curated, reused, and re-hafted many times before being discarded or lost corroborating Spithoven’s earlier interpretation of the life cycle of the small points [29, p. 89]. The bone material accommodated this intense reuse, but material selection, e.g., human and brown bear bones, may also suggest that these points were imbued with specific cultural and symbolic connotations [26].

There are errors in the caption for Fig 8. Please see the complete, correct Fig 8 caption here.

**Fig 8:**
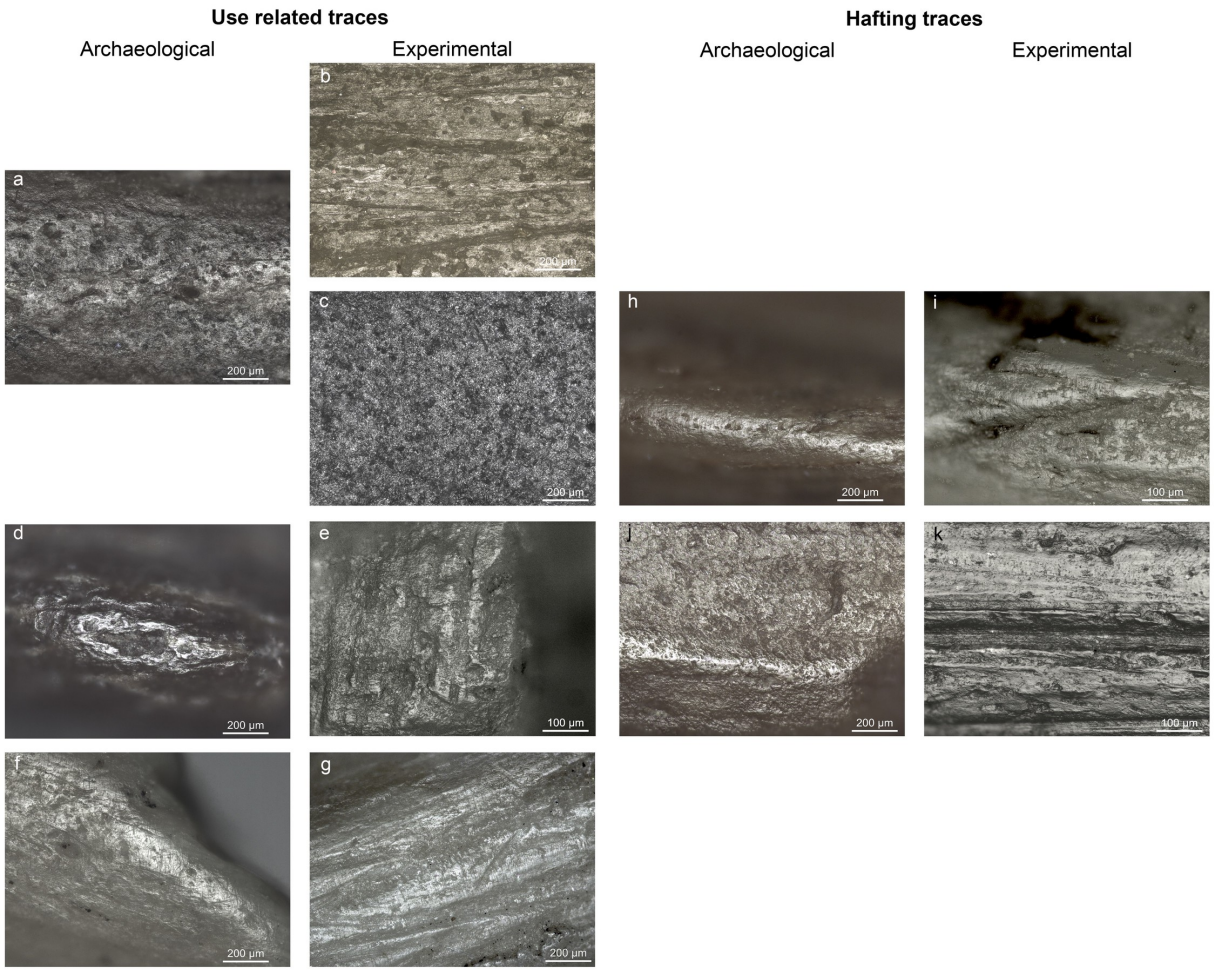
Comparison between archaeological and experimental wear traces. a) fish polish on NSM07 (100x); b) polish on an experimental bone point from shooting salmon (100x)*; c) polish on an experimental flint tool used to process fish (red snapper) d) bone polish on the second barb of NSM22 (100x); e) bone polish on an experimental point used to shot a carcass (100x)*; f) polish and short transverse striations on NSM17 from boring animal skin (100x); g) polish and short transverse striations on an experimental borer used to perforate deer skin (100x); h) polish and short transverse striations on the base of NSM08 from sinew bindings (100x); i) polish and short transverse striations from sinew bindings (200x); j) smooth bright polish on the base of NSM29 from plant bindings (100x); k) flat polish from lime bast bindings (200x). *Experiments conducted by Spithoven.

The following information is missing from the Acknowledgments section: We are grateful to Merel Spithoven-Stikkelorum, University of Groningen (NL), for her invaluable help in contacting the amateurs. Her 2018 master thesis on the Doggerland bone points constituted an inspiration for this study. She is currently extending this work in her PhD research in the context of the project Resurfacing Doggerland (NWO AIB19.009) directed by Dr. Hans Peeters, University of Groningen (NL).
