# Supplementary material for: The dynamic lives of osseous points from Late Palaeolithic/Early Mesolithic Doggerland: A detailed functional study of barbed and unbarbed points from the Dutch North Sea
Source: PLoS One. 2023 Aug 2;18(8):e0288629. doi: 10.1371/journal.pone.0288629 (PMC10395991; doi:10.1371/journal.pone.0288629)
Supplement: S1 Fig — (DOCX) [file pone.0288629.s001.docx]

**Supporting Information for “The dynamic lives of osseous points from Late Palaeolithic/Early Mesolithic Doggerland”**

A. Aleo, P.R.B. Kozowyk, L.I. Baron, A.L. van Gijn, G.H.J. Langejans

Corresponding author: Alessandro Aleo [a.aleo@tudelft.nl](mailto:a.aleo@tudelft.nl)

**S4 Figures**


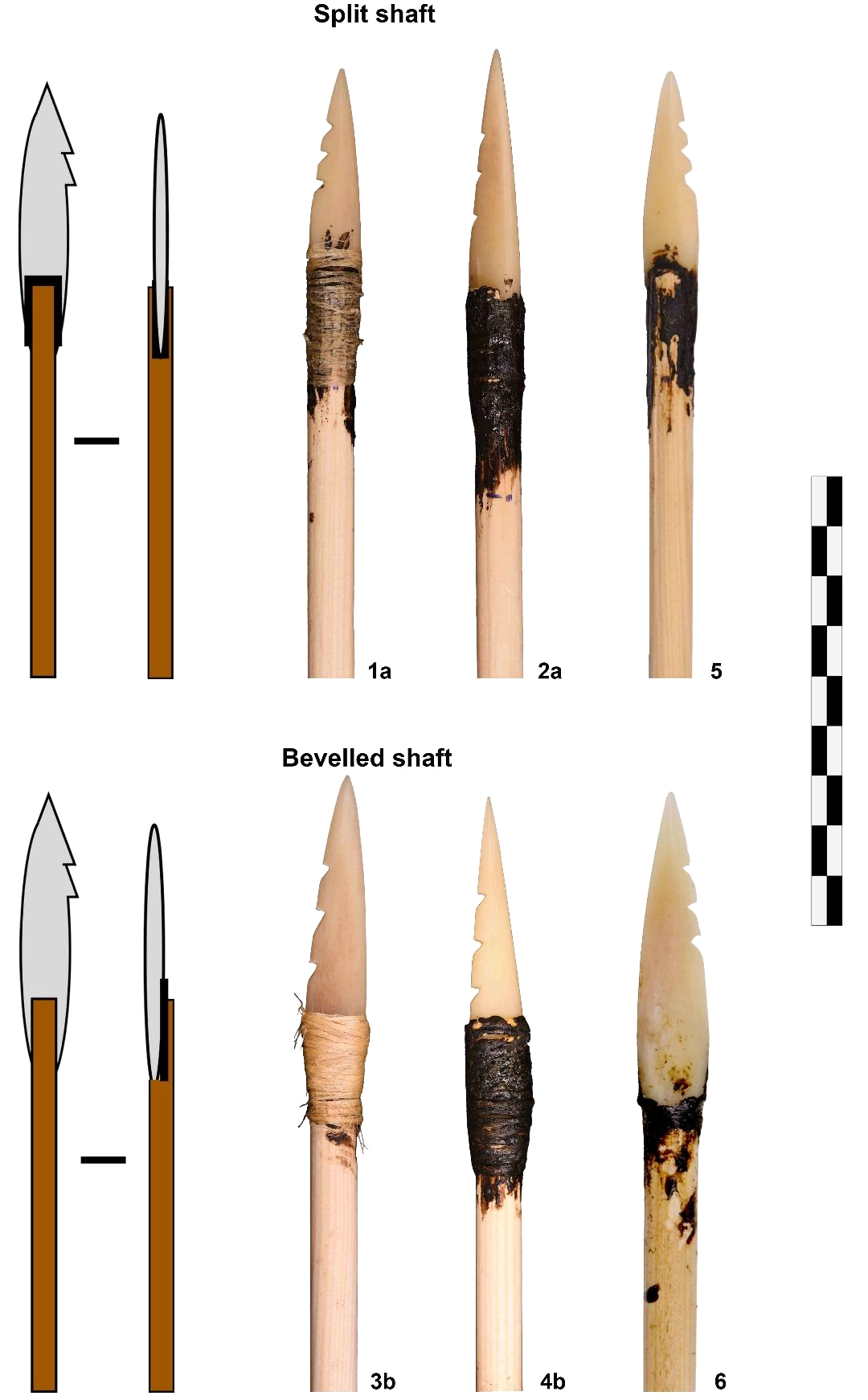


Fig 1: Different hafting designs tested during the experiment.


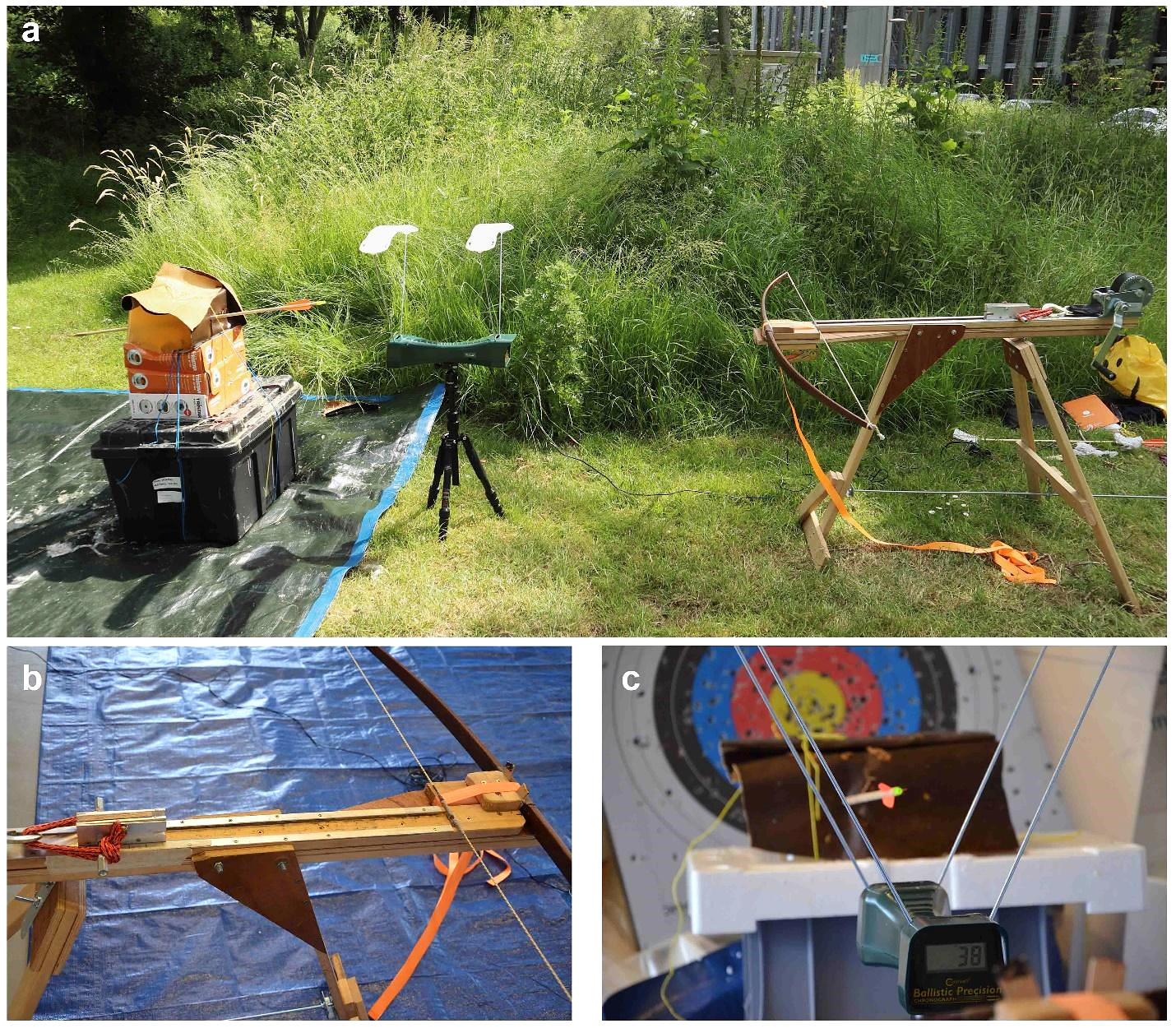


Fig 2: a) bone points experiment set-up; b) detail of the shooting machine; c) detail of the chronograph used to record arrows speed.
