# Supplementary material for: The dynamic lives of osseous points from Late Palaeolithic/Early Mesolithic Doggerland: A detailed functional study of barbed and unbarbed points from the Dutch North Sea
Source: PLoS One. 2023 Aug 2;18(8):e0288629. doi: 10.1371/journal.pone.0288629 (PMC10395991; doi:10.1371/journal.pone.0288629)
Supplement: S1 Table — (DOCX) [file pone.0288629.s002.docx]

**Supporting Information for “The dynamic lives of osseous points from Late Palaeolithic/Early Mesolithic Doggerland”**

A. Aleo, P.R.B. Kozowyk, L.I. Baron, A.L. van Gijn, G.H.J. Langejans

Corresponding author: Alessandro Aleo [a.aleo@tudelft.nl](mailto:a.aleo@tudelft.nl)

**S3 Tables**

Table 1: Morphometric data of experimental arrows. For experiments 17 and 18 the same point was used twice (a and b).

| **Exp nr** | **Point length (mm)** | **Point width (mm)** | **Point thickness (mm)** | **Point weight (mm)** | **Shaft spine test** | **Arrow length (mm)** | **Arrow weight (g)** |
| --- | --- | --- | --- | --- | --- | --- | --- |
| 1 | 65 | 11 | 4 | 3.72 | 0,59 | 883 | 32.68 |
| 2 | 67 | 12 | 4 | 4.48 | 0,45 | 881 | 37.76 |
| 3 | 68 | 12 | 5 | 4.99 | 0,54 | 893 | 33.91 |
| 4 | 65 | 11 | 5 | 3.79 | 0,41 | 888 | 35.86 |
| 5 | 61 | 10 | 4 | 3.21 | 0,46 | 884 | 36.473 |
| 6 | 63 | 11 | 4 | 3.74 | 0,45 | 885 | 36.19 |
| 7 | 66 | 13 | 5 | 4.83 | 0,45 | 888 | 35.84 |
| 8 | 69 | 11 | 5 | 4.80 | 0,49 | 892 | 36.07 |
| 9 | 64 | 10 | 5 | 4.01 | 0,46 | 883 | 35.62 |
| 10 | 66 | 10 | 5 | 4.20 | 0,49 | 887 | 36.08 |
| 11 | 66 | 10 | 5 | 4.59 | 0,52 | 885 | 33.49 |
| 12 | 56 | 10 | 5 | 3.54 | 0,43 | 879 | 38.15 |
| 13 | 64 | 12 | 5 | 4.20 | 0,56 | 893 | 36.24 |
| 14 | 51 | 11 | 5 | 3.11 | 0,40 | 873 | 38.89 |
| 15 | 58 | 11 | 4 | 3.03 | 0,45 | 877 | 38.69 |
| 16 | 59 | 11 | 5 | 3.64 | 0,54 | 881 | 36.03 |
| 17a | 55 | 11 | 4 | 3.54 | 0,42 | 876 | 39.1 |
| 17b | 55 | 11 | 4 | 3.54 | 0,43 | 875 | 38.69 |
| 18a | 65 | 12 | 5 | 4.42 | 0,54 | 885 | 33.18 |
| 18b | 65 | 12 | 5 | 4.42 | 0,54 | 885 | 33.23 |

Table 2: Summary of results of the ballistic experiment with replicas of bone points. For experiments 17 and 18 the same point was used twice (a and b). Experiments 3, 5, 6 and 8 were lost during shooting. M.D.= missing data; N.A.= not applicable.

| **Exp nr** | **Hafting type** | **N of shots** | **Re-hafting** | **Reasons for re-hafting** | **Av speed (m/s)** | **SD** | **Hating traces** | **Residue distribution** |
| --- | --- | --- | --- | --- | --- | --- | --- | --- |
| 1 | 3a | 25 | 2 | Arrow was not flying straight.  1) Shaft broke because of the impact angle  2) Point came off after hitting the styrofoam | 41 | 1.41 | - Greasy rough polish on lateral edges and dorsal face  - Discolouration on ventral face | Mostly on the ventral face; a few on lateral edges |
| 2 | 4a | 25 | 1 | Adhesive not curated enough | 39.8 | 1.5 | - Greasy rough polish on lateral edges and dorsal face  - Light discolouration at haft limit | Dorsal and ventral face mesial (haft limit) |
| 3 | 3b | 5 | 0 |  | M.D. | M.D. | N.A. | N.A. |
| 4 | 1b | 24 | 0 |  | 37.5 | 2.59 | - Flat smooth polish on lateral edges (especially left one). A few spots with transverse directionality  - Light discolouration | Dorsal and ventral face and lateral edges |
| 5 | 2b | 5 | 0 |  | 33.5 | 0.7 | N.A. | N.A. |
| 6 | 3b | 5 | 1 |  | 38 | 2.94 | N.A. | N.A. |
| 7 | 3a | 25  25 | 2 | 1) Adhesive not curated enough  2) Re-hafted the other way around | 39.78  39.16 | 2.74  2.12 | - Greasy polish dorsal and ventral face and lateral edges. A few spots on the edge with transverse directionality | Dorsal and ventral face. Very few on lateral edges |
| 8 | 4b | 4 | 0 |  | 39.25 | 1.5 | N.A. | N.A. |
| 9 | 2a | 18 | 1 | Arrow was not flying straight. Stuck in the styrofoam | 34.83 | 2.63 | - Binding discolouration (parallel bands) lateral left edge  - Greasy polish on lateral edges, no directionality | A few dorsal and ventral face mesial (haft limit) |
| 10 | 1a | 25 | 0 |  | 39.06 | 0.57 | - Greasy polish on lateral edges  -Smooth domed polish mesial ventral face (haft limit) | Dorsal and ventral face. Fewer on lateral edges |
| 11 | 2a | 20 | 0 |  | 40.88 | 0.6 | - Greasy dull polish on lateral edges with transverse directionality | A few dorsal and ventral face mesial (haft limit) |
| 12 | 1a | 25 | 0 |  | 36 | 1.86 | - Greasy polish on lateral edges with transverse directionality  - Smooth domed polish mesial ventral face (haft limit) | Dorsal and ventral face. Fewer on lateral edges |
| 13 | 4a | 25 | 0 |  | 38.45 | 2.01 | - Binding discolouration (parallel bands) on dorsal face  - Greasy rough polish on lateral edges and dorsal face | One little residue mesial (haft limit) |
| 14 | 4b | 2 | 0 |  | 37.5 | 2.12 | - No traces | Dorsal and ventral face mesial (haft limit) |
| 15 | 2b | 5 | 0 |  | 37 | 1.41 | - Light discolouration at haft limit | Dorsal and ventral face mesial (haft limit) |
| 16 | 1b | 18 | 0 |  | 37 | 1.63 | - No traces on the lateral edges | Dorsal, ventral and lateral edges |
| 17a | 5 | 11 | 0 |  | 40.75 | 3.77 | - No traces | Dorsal ventral face and a few on the lateral sides |
| 17b | 5 | 9 | 0 |  | 41.5 | 3.53 | - No traces | Dorsal ventral face and a few on the lateral sides |
| 18a | 6 | 1 | 0 |  | 38 | N.A. | - No traces | Ventral face |
| 18b | 6 | 1 | 0 |  | 37 | N.A. | - No traces | Ventral face |
