# Supplementary material for: The dynamic lives of osseous points from Late Palaeolithic/Early Mesolithic Doggerland: A detailed functional study of barbed and unbarbed points from the Dutch North Sea
Source: PLoS One. 2023 Aug 2;18(8):e0288629. doi: 10.1371/journal.pone.0288629 (PMC10395991; doi:10.1371/journal.pone.0288629)
Supplement: S1 File — (DOCX) [file pone.0288629.s003.docx]

**Supporting Information for “The dynamic lives of osseous points from Late Palaeolithic/Early Mesolithic Doggerland”**

A. Aleo, P.R.B. Kozowyk, L.I. Baron, A.L. van Gijn, G.H.J. Langejans

Corresponding author: Alessandro Aleo [a.aleo@tudelft.nl](mailto:a.aleo@tudelft.nl)

**S1 Destructive analysis – Expanded methodology**

*^14^C-AMS dating*

Two residue samples were analysed at Centre for Isotope Research (CIO), University of Groningen (NL) for radiocarbon dating. For a detailed description of the method see [1]. The submitted sample material from point 1 was pre-treated with acid only (‘A’). The material seemed to be too vulnerable and too small in size to do additional chemical pre-treatment steps (base and a second acid step). Sample from point 18 was pre-treated according to the ABA-protocol. The base-step was performed at room temperature instead of 80°C since tar/resin can be vulnerable when treated with alkaline solution at higher temperature.

*Gas chromatography – mass spectrometry (GC-MS)*

Three residue sample (tar/resin) were analysed at Inorganic Systems Engineering (ISE) Lab, TU Delft (NL) by gas chromatography-mass spectrometry (GCMS). Up to 10 milligrams of each material manufactured were sampled with a sterile scalpel blade and ground. Dichloromethane (DCM) HPLC grade purchased from TCI was added to obtain around 4 mg mL−1 solution. Extraction was done by ultrasonication for 30 minutes. An aliquot of the supernatant 200 µL was transferred to a 2mL glass vial with an insert, evaporated until dryness under a gentle stream of nitrogen. Derivatisation was made using bis(trimethysilyl)trifluoroacetamide (BSTFA) containing 1% trimethylchlorosilane purchased from Sigma–Aldrich. Ten microlitres of DCM, 5 µL of pyridine and 50 µL of BSTFA were added to the dry extract and the reaction took place for 20 min at room temperature. It was then evaporated to dryness under nitrogen stream at 30°C and recovered with DCM. One microlitre of the sample was then injected into the GCMS.

The GC-MS analyses were performed on an Agilent 7890B gas chromatograph system with an split/splitless inlet, coupled with an Agilent 5977B EI MSD interface, an FID and a splitter with corresponding EPC pressure control to achieve this. The GC was fitted with a nonpolar Agilent J&W DB5 MS column (30 m × 0.25 mm i.d.; 0.25 μm film thickness). The samples were introduced in a split/splitless injector used in the splitless mode at 300 °C with septum purge flow 3ml/min. The oven temperature was held isothermally for 2 min at 50 °C and ramped at 10 °C per minute to 150 °C and ramped at 4°C per minute to 320 °C and was held at that temperature for 20 min. The analysis was carried out using helium as carrier gas at a constant flow rate of 1.6 mL/min (average velocity 32.146 cm/sec). The temperature of FID was set at 340 °C and hydrogen flow was 30 ml/min, synthetic air 400 ml/min, nitrogen 30 ml/min. The temperatures of the ion source were set at 230 °C and the transfer line at 280 °C. The mass spectrometer was monitored to scan 35–950 m/z with an ionizing voltage at 70 eV. Total run time was 75 min and solvent delay was 4 min. GC–MS chromatogram was interpreted using National Institute standard and Technology (NIST). The mass spectra were matched against those of authentic standards (betulin and lupeol), by using previously published data and the NIST library.

*Zooarchaeology by mass spectrometry (ZooMS)*

Three bone samples were analysed at BioArch, University of York (UK) for ZooMS by using the ammonium bicarbonate protocol developed by Doorn et al. [2]. The non-destructive buffer extraction was opted because of the small samples size. The samples were suspended in 50 mM ammonium bicarbonate (ABC) and left overnight at room temperature. The samples were then centrifuged and the supernatant discarded. This was to remove any surface contamination. The samples were then resuspended in 50mM ABC, heated at 65° for one hour to gelatinise any available collagen into solution and the supernatant was collected. 1 μL of enzyme trypsin (Promega) was added to this and the samples were digested overnight at 37°. The digestion was stopped the following morning with the addition of 5% TFA (trifluoroacetic acid). This strong acid kills off the enzyme. The samples were purified using zip-tips and eluted in 100 μL 50:50 0.1%TFA in dH2O: ACN.

The samples were spotted in triplicate onto a MALDI plate and a matrix was added to each sample spot (α-cyano-4-hydroxycinnamic acid (CHCA)). The samples were run on a Bruker ultraflex MALDI-ToF instrument. The three replicates were averaged and the identifications were made by comparing the peaks to a list of published peptide marker series for all European, Pleistocene medium to large size mammals [3].

1. Dee MW, Palstra SWL, Aerts-Bijma AT, Bleeker MO, de Bruijn S, Ghebru F, et al. Radiocarbon Dating at Groningen: New and Updated Chemical Pretreatment Procedures. Radiocarbon. 2020;62(1):63-74.

2. Van Doorn NL, Hollund H, Collins MJ. A novel and non-destructive approach for ZooMS analysis: ammonium bicarbonate buffer extraction. Archaeological and Anthropological Sciences. 2011;3(3):281.

3. Welker F, Hajdinjak M, Talamo S, Jaouen K, Dannemann M, David F, et al. Palaeoproteomic evidence identifies archaic hominins associated with the Châtelperronian at the Grotte du Renne. Proceedings of the National Academy of Sciences. 2016;113(40):11162.
