# Supplementary material for: The dynamic lives of osseous points from Late Palaeolithic/Early Mesolithic Doggerland: A detailed functional study of barbed and unbarbed points from the Dutch North Sea
Source: PLoS One. 2023 Aug 2;18(8):e0288629. doi: 10.1371/journal.pone.0288629 (PMC10395991; doi:10.1371/journal.pone.0288629)
Supplement: S2 File — (DOCX) [file pone.0288629.s004.docx]

**Supporting Information for “The dynamic lives of osseous points from Late Palaeolithic/Early Mesolithic Doggerland”**

A. Aleo, P.R.B. Kozowyk, L.I. Baron, A.L. van Gijn, G.H.J. Langejans

Corresponding author: Alessandro Aleo [a.aleo@tudelft.nl](mailto:a.aleo@tudelft.nl)

**S2 Extended results**

*3D models of bone points with mastic and hafting traces*

3D models can be viewed and downloaded from 4TU.ResearchData repository.

Doi: 10.4121/8ecbf77d-5442-4447-8761-2c0c14393b0f

*^14^C-AMS dating*

Direct dating of NSM1.2 sample of the tar yielded a ^14^C date of 9275±35 BP (GrM-27499).
The δ^13^C value of the tar is -28.42‰, and the organic carbon content 62.7%. Both are
normal values for this material [1]. The date obtained for the tar is calibrated to calendar years with OxCal, version 4.4 [2] using IntCal20 calibration curve [3]. We obtained an absolute age of 10573-10298 years BP.

Direct dating of NSM18.2 sample of the tar yielded a ^14^C date of 11065±50 BP (GrM-27889).
The δ1^3^C value could not be calibrated well for sample NSM18.2 in the specific combustion batch,

because the amount of carbon in the sample was too low (carbon content <1). The date obtained for the tar is calibrated to calendar years with OxCal, version 4.4 [2] using IntCal20 calibration curve [3]. We obtained an absolute age of 13095-12843 years BP.

1. Mook WG. International Comparison of Proportional Gas Counters for 14C Activity Measurements∗. Radiocarbon. 1983;25(2):475-84.

2. Bronk Ramsey C. Bayesian Analysis of Radiocarbon Dates. Radiocarbon. 2009;51(1):337-60.

3. Reimer PJ, Austin WEN, Bard E, Bayliss A, Blackwell PG, Bronk Ramsey C, et al. The IntCal20 Northern Hemisphere Radiocarbon Age Calibration Curve (0–55 cal kBP). Radiocarbon. 2020;62(4):725-57.
